# Quantification of Reaction Barriers Under Diffusion Controlled Conditions

**DOI:** 10.1002/jcc.70233

**Published:** 2025-09-27

**Authors:** Martin M. Maehr, Radu A. Talmazan, Maren Podewitz

**Affiliations:** ^1^ Institute of Materials Chemistry, TU Wien Vienna Austria

## Abstract

In quantum chemistry, diffusion‐controlled reactions are typically characterized by a monotonous rise in the electronic energy, indicative of a barrierless process. In reality, this change in electronic energy is accompanied by an increase in entropy, thereby introducing a barrier in free energy. Standard quantum‐chemical models fall short in capturing this phenomenon, but we have developed a cost‐efficient method to address this challenge. By tracking changes in bonding based on quantum chemical descriptors, we can model the onset of entropy along the reaction path by defining a cutoff that indicates the halfway point in the entropy gain. Utilizing a sigmoid fit function to model the entropy change, we obtain a transition state on the free energy surface for diffusion‐controlled reactions. Our methodology is robust and suitable for diverse complexes within both organic and inorganic chemistry.

## Graphical abstract

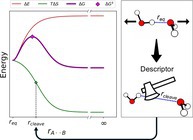

## 1 Introduction

The framework of static, state‐of‐the‐art quantum chemistry lacks a straightforward way of calculating the onset of entropy in (barrierless) reactions, characterized by a monotonous increase in electronic energy with growing fragment separation.

However, in reality, a barrier in the Gibbs free energy along the reaction path appears, as the two fragments start to move independently, accompanied by an increase in translational and rotational entropy. From an experimental point of view, these reactions can be understood to be controlled by diffusion. In theory, such transition states are also referred to as “loose” transition states and are characterized by a free or almost free rotation of the two fragments with respect to each other [1]. Most accurately, the free energy would be obtained from repeated sampling of the reaction path using ab initio molecular dynamics and subsequent entropic path sampling. However, this strategy is computationally very demanding even for medium‐sized molecules [2]. Thus, finding appropriate solutions to adequately approximate the entropy in chemical reactions remains one of the challenges in computational chemistry [3, 4].

In modern quantum chemical methodologies, the thermal and entropic contributions are calculated by approximating the partition function q, translating a single molecule into an ensemble at finite temperature and pressure,

(1) $$q=\sum_{i}^{\infty }g_{i}e^{\frac{-\varepsilon_{i}}{\mathrm{kT}}}$$

with $\varepsilon_{i}$ being the energy of the microstate i and $g_{i}$ being the degeneracy factor. The partition function q is separated into rotational, translational, vibrational and electronic contributions, assuming an ideal gas, rigid rotor, and harmonic oscillator model (RRHO). The resulting q can be used to calculate the entropy,

(2) $$S=\mathrm{kT}{\left(\frac{\partial \ln q}{\partial T}\right)}_{V}+k\ln q$$

To quantify entropic changes calculated with this standard model, Watson and Eisenstein showed that for typical organic reactions the entropy gain associated with the splitting of a molecule into two fragments, is about $35\;\mathrm{kJ}\;{\mathrm{mol}}^{-1}$, mostly accounted for by changes in translational and rotational entropy, with minor contributions from vibrational entropy [5]. While the RRHO model predominates in quantum chemistry, there are inherent limitations due to the nature of the approximated partition function [6]. Efforts have been made to improve the description of the translational entropy by introducing the free volume, taking into account the accessible volume of a molecule in the condensed phase [7, 8, 9]. The vibrational entropy relies on the calculation of harmonic frequencies, which are increasingly incorrect for low‐modes with < 100 ${\mathrm{cm}}^{-1}$. To correct for this shortcoming, low lying modes can be either be described by a free rotor model [10] or shifted to 100 ${\mathrm{cm}}^{-1}$ [11]. In addition, all frequencies are often scaled by a constant factor to further increase accuracy [12]. These estimates are commonly referred to as quasi‐harmonic approximations. In recent years, the RRHO model has been augmented to account for conformational entropy, derived from conformational sampling tools [13, 14] or from classical molecular dynamics simulations [15, 16].

As the RRHO model, in conjunction with transition‐state theory (TST), can only account for translational and rotational contributions of the system as a whole, it does not allow for the onset of rotation and translation of the fragments upon separation, and it can typically only deal with stationary points on the potential energy surface (PES) (see Figures S6 and S7). A notable exception to the latter is the Single Point Hessian method by Spicher and Grimme that allows for the evaluation of the vibrational frequencies, through the Hessian, at non‐equilibrium points by employing a biasing constraining potential to fix the initial geometry [17]. Another strategy to calculate the vibrational entropy at non‐stationary points along the reaction path is the frequency projection method, which ensures the orthogonality of the vibrational frequencies with respect to the reaction path and rotational motions [18, 19, 20]. However, this requires the usage of curvilinear coordinates to obtain meaningful results and avoid discontinuities in the free energy [1]. Upon separation of the fragments, the vibrational entropy becomes less favorable (and decreases) as six vibrational degrees of freedom merge into three rotational and three translational degrees of freedom. Once these vibrational frequencies fall below a threshold, they must be treated as rotations and translations. While both the frequency projection and the Single Point Hessian methods account for the decrease in vibrational entropy, only the latter correctly describes the low modes through a (hindered) rotor approximation [10].

Furthermore, even if vibrational entropy is treated correctly and emerging rotational modes are described by a hindered rotor, the RRHO model is still not able to account for the onset of translational entropy and only approximates rotational entropy. In addition, the Hessian matrix computations required to gain multiple configurations along the reaction path remain costly, despite recent efforts aiming for a computation at a lower level of theory [17].

In an attempt to extend conventional TST, where the transition state is identified as the highest energy point of the minimum energy path on the PES, variational transition state theory (VTST) [21] was developed. Here, the transition state is determined by variationally optimizing the dividing surface between educts and products so that the net reaction flux is minimized.

While VTST has originally been developed for systems with a well‐defined (or “sharp”) barrier on the PES, it can be extended to “loose” transition states, using the variable‐reaction‐coordinate variational transition‐state theory (VRC‐VTST) [22, 23]. Loose transition states are characterized as difficult to localize on a PES, and their dividing surface does not correspond to a saddle point on the PES; hence, their barriers are dominated by entropic contributions [24]. However, VRC‐VTST calculations require massive Monte Carlo sampling and are therefore very expensive [22].

Due to these computational limitations and the frequent occurrence of diffusion‐controlled reaction steps in chemistry, an alternative approach has been developed: Some years ago, McMullin et al. encountered a ligand dissociation for a palladium complex [25]. The free energy barrier associated with this reaction step has been experimentally estimated to approach the diffusion limit, corresponding to approximately $20\;\mathrm{kJ}\;{\mathrm{mol}}^{-1}$ [25, 26] in most common solvents. This value has widely been accepted as a rule of thumb barrier for such reactions.

**FIGURE 1:**
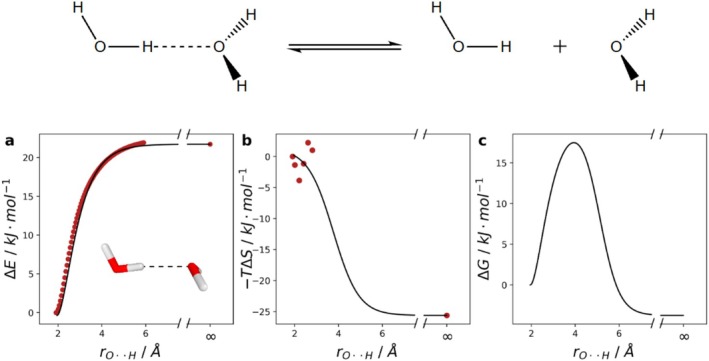
Entropy estimation according to Ryu et al. [27], demonstrated on the dissociation of a water dimer, depicted above. (a) Scanned electronic energy (red dots) and fitted morse‐potential function (black line). (b) Entropic contributions calculated using RRHO around the equilibrium bond distance (red dots) and fitted sigmoidal function (black line). (c) Gibbs free curve, yielded through summation of the electronic and entropic fit‐functions in (a) and (b) (black line). Values were reproduced according to the methodology of Ryu et al. using B3LYP/def2‐TZVP.

In a more rigorous approach than simply considering the barrier to reflect the dissociation limit, Ryu et al. estimated the onset of entropy by interpolating the values between a complex, A‐B, and the two separated fragments A and B (see Figure 1) [27].

The electronic energy is scanned in incremental steps, while elongating the bond distance between the hydrogen‐oxygen bridge ($r_{O\cdot \cdot H}$). A morse‐potential function is then fitted through the data points, with an additional point given at infinite separation, with its magnitude set to the electronic energy of two isolated water molecules (Figure 1a). The entropic contributions −TΔS are modeled by computing the entropy for the equilibrium structure and structures with small elongation of $r_{O\cdot \cdot H}$. The entropic contributions at infinite separation are added to the dataset and a sigmoidal function is fitted (Figure 1b). By adding up the electronic and the entropic fit functions, the estimated Gibbs free energy surface for the water dimer (c) is obtained.

However, this approach still has several drawbacks. First of all, it requires the calculation of the entropic corrections (second order derivatives) for multiple conformations along the reaction path, which becomes computationally very costly, especially for large molecules. Second, the obtained entropy data points get increasingly incorrect as the dissociation progresses from the energy minimum. Third, the fit of the sigmoid function is ambiguous: (i) it is not clear at which point the entropy should have the maximum increase, that is, where the inflection point of the function is, and (ii) it is not clear how steep the sigmoid function should be. Our experience is also that the entropic contributions rarely decrease stringently upon bond length increase but often scatter (compare Figures S1 and S2) [28]. Consequently, the results depend on the chosen parameters.

Inspired by this work, we developed a rigorous scheme to estimate the onset of entropy in dissociation reactions, by addressing the aforementioned issues. We assumed that 50% of the final entropy is reached once the bond is broken and deduced the bond cleavage distance from analysis of chemical descriptors, such as bond order indices, calculated along the reaction path. This distance was set as the inflection point of our sigmoid‐shaped entropy fit function. The steepness of the function was determined by adjusting a parameter k, while assuming that there is no energy minimum after the inflection point. We developed our methodology at the example of the water dimer, testing a set of quantum chemical descriptors, benchmarking against experimental data. Comparison with high‐level computational (VTST) reference data for dissociation of non‐covalently bound S_*N*_2 encounter complexes, as well as covalently bound complexes, yielded very good agreement of our methodology. In addition to these gas phase reactions, the applicability of our approach to large inorganic complexes in implicit solvent has also been demonstrated.

## 2 Computational Methodology

All quantum chemical calculations were done using density functional theory (DFT) and were performed using the program ORCA 5.0.4 [29]. The geometry of the initial and completely dissociated species were fully optimized, while dissociations were performed through a relaxed scan with a step size of 0.05 Å. The water dimer, S_*N*_2 type complexes, $\left\{{\mathrm{Cl}}^{-}{\mathrm{CH}}_{3}X\right\}$ with X = [Cl, Br] as well as ethylamine and $C_{2}F_{4}$ are all described with B3LYP [30], the def‐TZVP basis set [31] and empirical dispersion corrections in the gas phase, in agreement with experimental or computational reference data. To account for the homolytical cleavage of the C–C bond in ethylamine, a Broken Symmetry approach was to use to obtain a singlet open shell wave function. While the initial water dimer study was performed with D3 corrections [32], all other studies were performed with D4 corrections [33]. However, the impact on the resulting barriers is negligible (see Figure S5). An extensive benchmark of the computational methodology, in addition to a detailed comparison of dispersion corrections for the water dimer, is found in the Supporting Information (Section 2), including Coupled Cluster (CCSD(T)) calculations with a def2‐QZVP basis set [31], while in the main manuscript only B3LYP results are shown for consistency. Comparison with Coupled‐Cluster reference calculations showed the difference between the electronic energies of CCSD(T) and B3LYP/def2‐TZVP/D3 was max. $5\;\mathrm{kJ}\;{\mathrm{mol}}^{-1}$ (Figure S6), close to chemical accuracy. More relevant for us, however, the agreement in the estimated barrier was even better, with a deviation of less than $2\;\mathrm{kJ}\;{\mathrm{mol}}^{-1}$, (see Figure S7). The Pd and the Au complexes were optimized using the same B3LYP/def2‐TZVP/D4 methodology, but an implicit solvation model (CPCM) was used [34], in agreement with experimental data. All frequency calculations have been calculated with the same combination of settings as the corresponding optimized geometries. All modes with frequencies below $100\;{\mathrm{cm}}^{-1}$ have been shifted to $100\;{\mathrm{cm}}^{-1}$ using GoodVibes [11, 35]. Quantum chemical descriptors, such as Mulliken partial charges, bond order indices, were calculated using the wave‐function analysis tool Multiwfn [36]. All of the fitting functions have been computed using the least‐quares algorithm of the Python 3 package SciPy [37] and are available as a Jupyter‐Notebook (https://github.com/PodewitzLab/DORA.git).

## 3 Results and Discussion

### 3.1 Determination of Transition States in Barrierless Reactions

**FIGURE 2:**
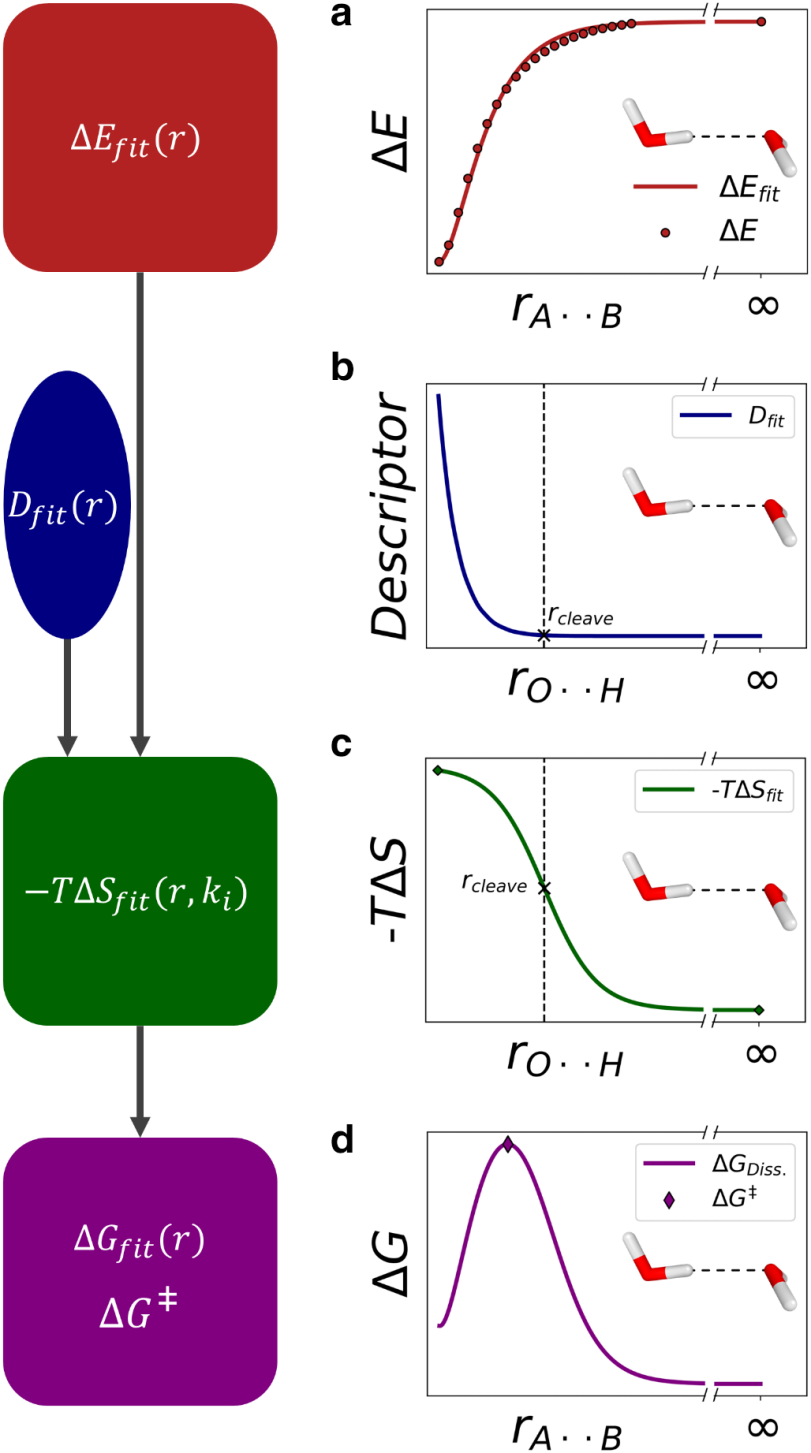
Schematic representation of the workflow for entropy estimation. (a) Calculation of the electronic energy during the dissociation reaction and fit with $\Delta E_{\mathrm{fit}}\left(r\right)$ (red). (b) Calculation of descriptor and fit function $D_{\mathrm{fit}}$ (blue) to determine inflection point of the entropy curve. (c) Fitted entropy $-T\Delta S_{\mathrm{fit}}\left(r\right)$ (green). (d) Calculation of the final Gibbs free energy surface $\Delta G_{\mathrm{fit}}\left(r\right)$ by adding up $\Delta E_{\mathrm{fit}}\left(r\right)$ and $-T\Delta S_{\mathrm{fit}}\left(r\right)$. The maximum of $\Delta E_{\mathrm{fit}}\left(r\right)$ is the free energy dissociation barrier $\Delta G^{‡}$.

Our methodology to estimate the entropy of barrierless reactions is as follows: First, the electronic dissociation energy is calculated and a morse potential function is fitted (Figure 2a). The onset entropy of the dissociation process is modeled using quantum chemical descriptors. The chosen descriptor is used to locate the intramolecular distance, $r_{\text{cleave}}$, at which the bond is assumed to break (Figure 2b). We use $r_{\text{cleave}}$ as the inflection point of the entropy fit. At this bond‐breaking distance, we define the entropy as half of its total value. The onset of entropy is modeled with a sigmoid function, and the resulting entropy curve $-T\Delta S_{\mathrm{fit}}\left(r,k_{i}\right)$ (Figure 2c) is added to the electronic energy $\Delta E_{\mathrm{fit}}\left(r\right)$, to yield the Gibbs free energy surface $\Delta G_{\mathrm{fit}}\left(r\right)$ (Figure 2). To ensure no minimum exists beyond the transition state (TS), the steepness of the sigmoid is adjusted accordingly. The maximum of $\Delta G_{\mathrm{fit}}\left(r\right)$ corresponds to the Gibbs free energy barrier $\Delta G^{‡}$.

### 3.2 Methodological Framework

A structure optimization of the entire molecule, as well as of each fragment, is followed by a relaxed surface scan along the dissociating bond. Through these data points, a morse function $\Delta E_{\mathrm{fit}}\left(r\right)$ is fitted, to gain a continuous function that describes the electronic energy of dissociation.

(3) $$\Delta E_{\mathrm{fit}}\left(r\right)=\Delta E_{\mathrm{eq}.}\left(e^{-2a\left(r-r_{\mathrm{eq}.}\right)}-2e^{-a\left(r-r_{\mathrm{eq}.}\right)}\right)$$

Here, $\Delta E_{\mathrm{eq}.}$ is the electronic energy at equilibrium distance $r_{\mathrm{eq}.}$ and a is a constant factor.

For each step of the scan, a quantum chemical descriptor is calculated. For example, this could be a bond‐order parameter. After normalizing the values of the descriptor between 0 and 1, an exponentially decaying function $D_{\mathrm{fit}}$ is fitted

(4) $$D_{\mathrm{fit}}\left(r\right)=D_{0}e^{-kr}$$

with $D_{0}$ being the maximum value of the given descriptor and k being a constant. We assume a bond to be broken if the change in the descriptor is smaller than a cutoff threshold $\gamma_{\text{cleave}}$. This is done by investigating the first derivative of $D_{\mathrm{fit}}$ with respect to r.

(5) $$\frac{\partial D_{\mathrm{fit}}\left(r\right)}{\partial r}\leq \gamma_{\text{cleave}}$$

The determined bond distance $r_{\text{cleave}}$ is assumed to be the point, where 50% of the total entropy is reached. Hence, it is the inflection point of the sigmoid function.

To model the change in entropy during the dissociation, we define three data points to fit a sigmoid function: The first point is the equilibrium distance of the complex AB, $r_{\mathrm{eq}}$, where we have $-T\Delta S_{\mathrm{AB}}$, the second point is at infinite distance, that is, with infinitely separated fragments A and B, where we have $-T\left(S_{A}+S_{B}\right)$, the third point is the distance at which we assume the bond of the fragments AB to be broken, which is the inflection point of the sigmoid $r_{\text{cleave}}$, where the entropy amounts to $-\frac{1}{2}T\left(\Delta S_{A}+S_{B}\right)$. Hence, we may formulate the entropy at a given temperature T as

(6) $$-T\Delta S_{\mathrm{fit}}\left(r\right)=-\frac{T\left(\Delta S_{A}+\Delta S_{B}\right)}{1+e^{-k\left(r-r_{\text{cleave}}\right)}}$$

with k being the steepness of the entropy curve. We assume that r is equal to $r_{\mathrm{eq}}$ at the start of the fit. At this distance, we have an entropy of $-T\Delta S_{\mathrm{AB}}$.

By adding $\Delta E_{\mathrm{fit}}$ to the entropic contribution $-T\Delta S_{\mathrm{fit}}$, we yield the Gibbs free energy $\Delta G_{\mathrm{fit}}$ upon dissociation (Figure 2c)

(7) $$\Delta G_{\mathrm{fit}}\left(r\right)=\Delta E_{\mathrm{fit}}\left(r\right)-T\Delta S_{\mathrm{fit}}\left(r\right)$$

where the maximum indicates the reaction barrier $\Delta G^{‡}$.

**FIGURE 3:**
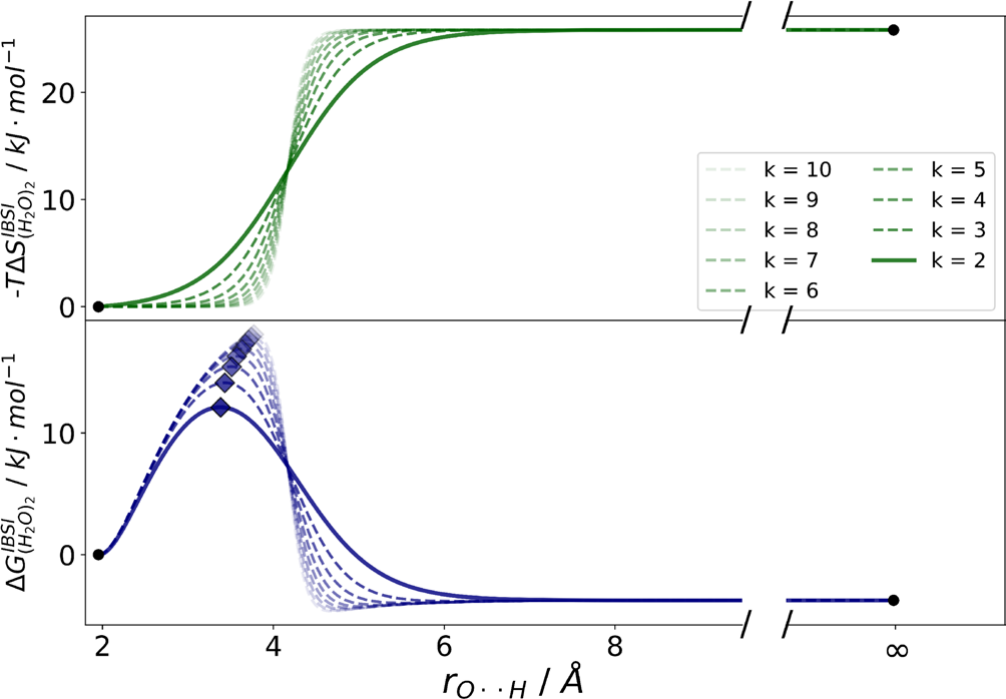
Iterative process to determine the steepness k of the entropy fit (green). The value k is lowered until the resulting free energy curve shows no minimum after the TS.

To estimate the optimal steepness of the sigmoid, we adjust the constant k (see Equation 6). High k values led to additional minima after the TS, (Figure 3). To avoid this, we iteratively decrease the k value, starting for example from k=10 until the resulting fit curve no longer exhibits a minimum after the TS.

### 3.3 The Water Dimer

**FIGURE 4:**
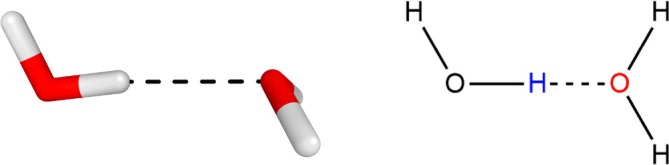
3D‐model (left) and Lewis structure (right) of our model system, the water dimer. The dashed line between indicates the bond to be dissociated.

To assess the outlined methodology to estimate the onset of entropy, we chose the water dimer as the model system (Figure 4). We calculated a set of quantum chemical descriptors along the bond dissociation, from equilibrium ($r_{\mathrm{eq}}=1.95$ Å) to 6 Å in steps of 0.05 Å, to test their ability to describe changes in the hydrogen bond. During this process, retention of the original orientation was ensured by the introduction of angle constraints.

**FIGURE 5:**
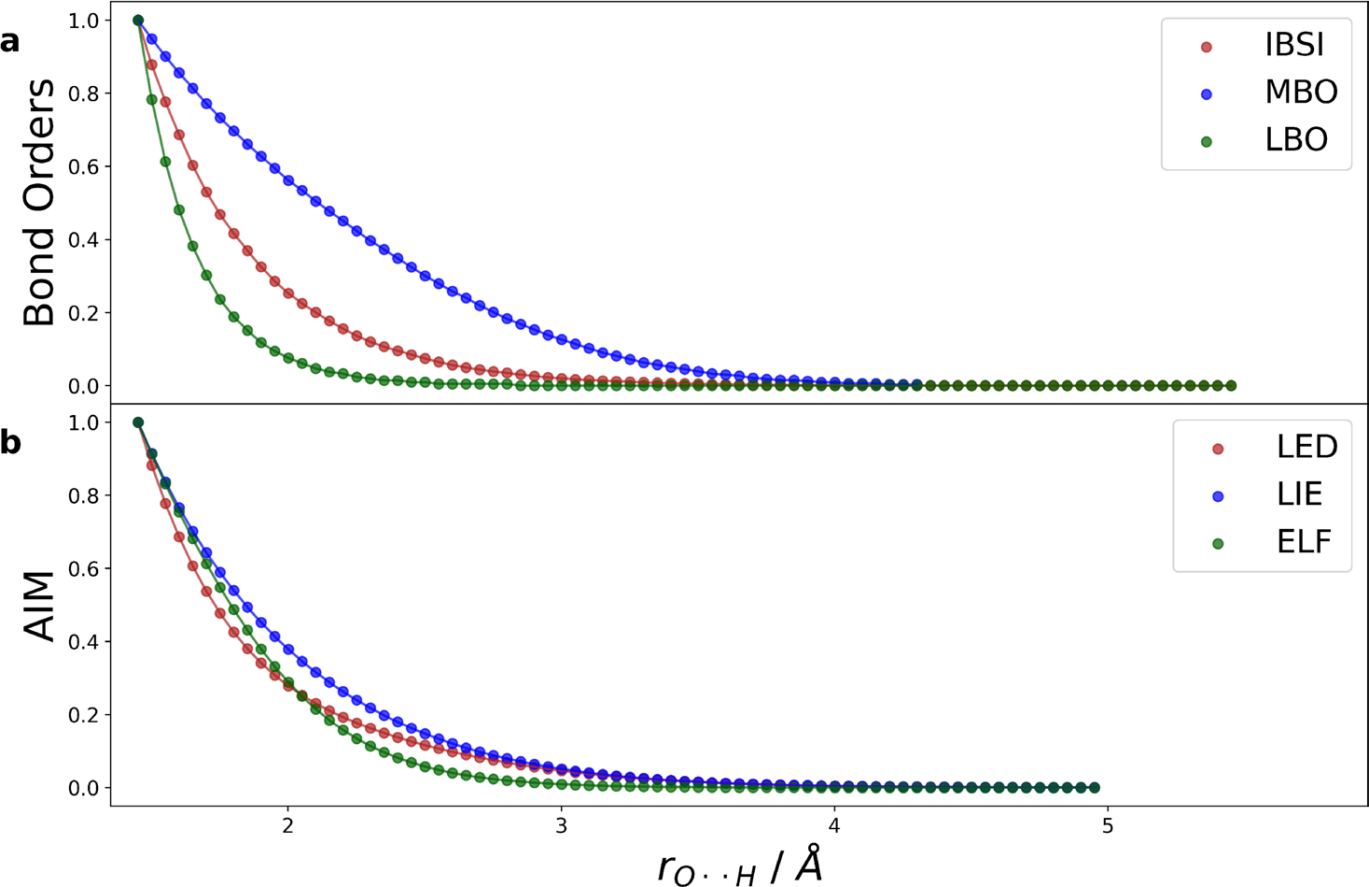
Quantum chemical descriptors along $r_{O\cdot \cdot H}$ bond dissociation of water dimer. (a) Bond orders; intrinsic bond strength (IBSI) [38], Mayer bond order (MBO) [39], and Laplacian bond order (LBO) [40]. (b) Descriptors gained from AIM [41] at the critical point between the bridging hydrogen and oxygen; Laplacian electron density (LED), local information entropy (LIE), and electron localization function (ELF).

Ideally, the descriptor to assess the bond cleavage should be computationally inexpensive. We showcase three bond order and three atoms‐in‐molecules (AIM) indices, while the remaining tested descriptors are discussed in the Figures S3 and S4. All descriptors were normalized (min, max) to [0, 1] and plotted against the intermolecular distance $r_{O\cdot \cdot H}$, as depicted in Figure 5.

Analyzing the results, they behave similarly, with an exponential decay of the descriptors as the bond length increases (see Figure 5). By fitting the values of the given descriptor, a continuous function is obtained, based on which we determine the bond cleavage point, that is, when the value of the slope becomes smaller than or equal to a threshold value $\gamma_{\text{cleave}}$ (see Equation 5).

**FIGURE 6:**
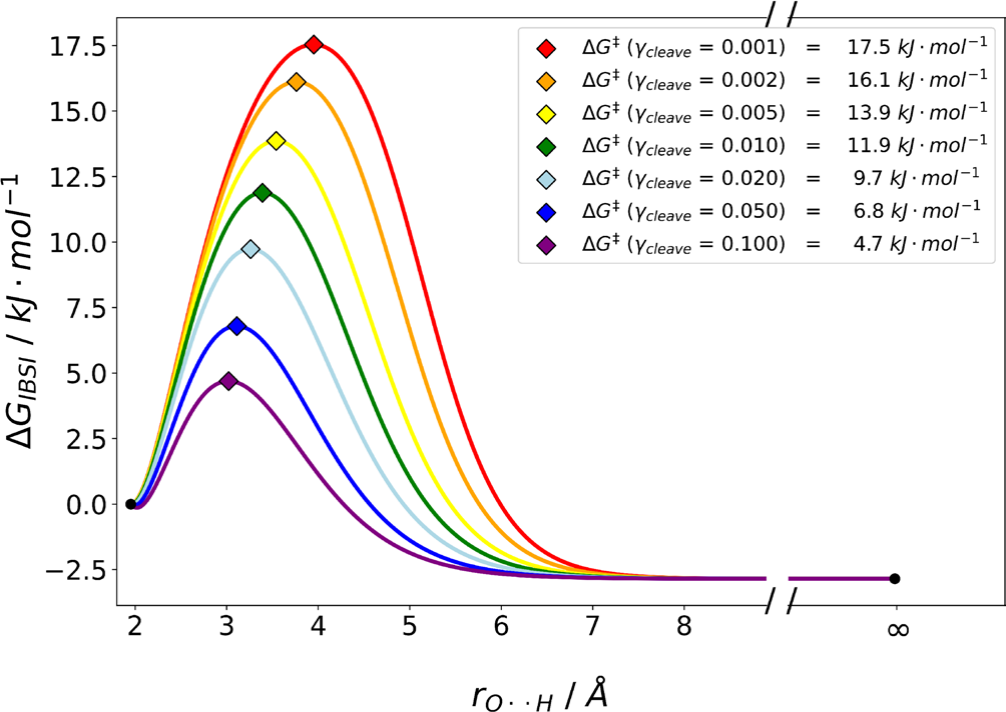
$\Delta G_{\mathrm{fit}}$ of the water dimer for different cutoff values $\gamma_{\text{cleave}}$ with the IBSI descriptor. The rainbow colored curves represent the free energy surfaces, while the diamonds indicate the location of the transition state. red: $\Delta G\left(\gamma_{\text{cleave}}=0.001\right)$; orange: $\Delta G\left(\gamma_{\text{cleave}}=0.002\right)$; yellow: $\Delta G\left(\gamma_{\text{cleave}}=0.005\right)$; darkgreen: $\Delta G\left(\gamma_{\text{cleave}}=0.010\right)$; turquoise: $\Delta G\left(\gamma_{\text{cleave}}=0.020\right)$; blue: $\Delta G\left(\gamma_{\text{cleave}}=0.050\right)$ violet: $\Delta G\left(\gamma_{\text{cleave}}=0.100\right)$. Results were obtained by B3LYP/def2‐TZVP with D3 dispersion corrections.

As it is not immediately apparent, what value for $\gamma_{\text{cleave}}$ should be used to define the breaking of the bond, we investigated its impact at the example of the intrinsic bond strength index (IBSI) descriptor. We determined the inflection point of −TΔS for a set of $\gamma_{\text{cleave}}$ values, testing different −TΔS fits. As a consequence, different $\Delta G_{\mathrm{fit}}$ curves and barriers $\Delta G^{‡}$ result. A set of free energy curves obtained with the IBSI descriptor is shown in Figure 6.

Clearly, the definition of the value of $\gamma_{\text{cleave}}$ has a big impact on the resulting energy barrier with values varying between $4.7\;\mathrm{kJ}\;{\mathrm{mol}}^{-1}$ (10% cutoff) and $17.5\;\mathrm{kJ}\;{\mathrm{mol}}^{-1}$ (0.1% cutoff).

**FIGURE 7:**
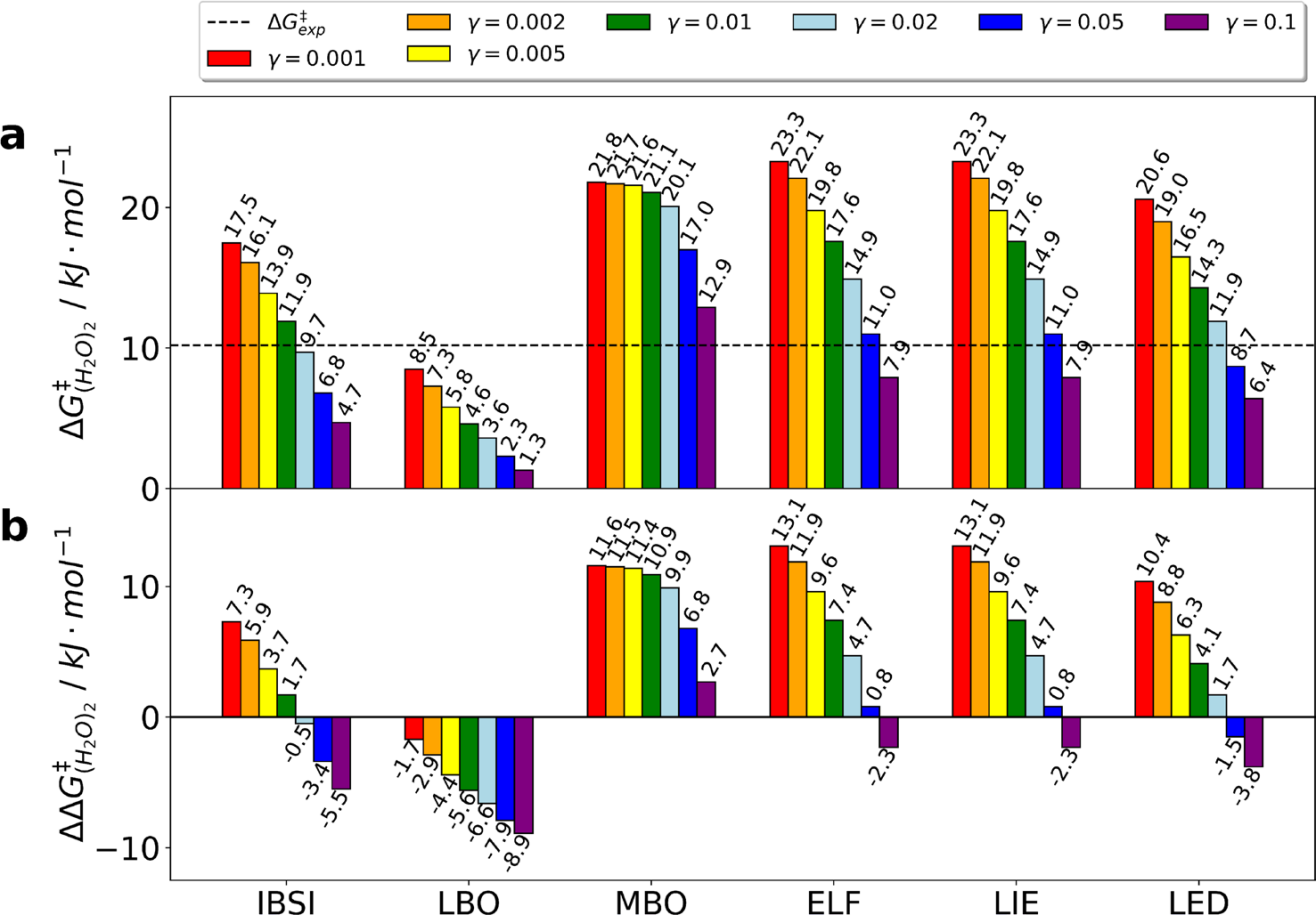
Barplot of the estimated dissociation barrier $\Delta G_{{\left(H_{2}O\right)}_{2}}^{‡}$ for various $\gamma_{\text{cleave}}$ values and quantum chemical descriptors (IBSI, LBO, MBO, ELF, LIE, LED). Values were obtained using B3LYP/def2‐TZVP with D3 dispersion corrections. (a) Comparison of absolute values; (b) comparison of differences to the experimental value of $\Delta G_{\exp}^{‡}=10.2\;\mathrm{kJ}\;{\mathrm{mol}}^{-1}$ [42].

Following the same procedure as for IBSI, we determined the dissociation barriers $\Delta G_{\text{diss}}^{‡}$ for all bond orders and AIM descriptors using the various $\gamma_{\text{cleave}}$ values (Figure 7a). The resulting values were compared to the experimentally determined (gas phase) barrier (Figure 7b) of $\Delta G_{\exp}^{‡}=10.2\;\mathrm{kJ}\;{\mathrm{mol}}^{-1}$ [42].

The analysis shows fairly consistent results with the exception of LBO, which yielded lower barriers (7, panel A). For all descriptors, the dissociation barriers for the water dimer become smaller with increasing $\gamma_{\text{cleave}}$ value. This is not surprising, since a higher value for $\gamma_{\text{cleave}}$ leads to a smaller bond breaking distance $r_{\text{cleave}}$. From that it follows that the entropy increases for smaller intramolecular distances, which counteracts the increasing electronic energy.

The IBSI descriptor yields the best agreement with the experimental results, with four out of seven $\gamma_{\text{cleave}}$ values deviating by less than $4\;\mathrm{kJ}\;{\mathrm{mol}}^{-1}$, being within the realm of chemical accuracy with respect to experiment. LBO and MBO show either under‐ or overestimation of the barrier up to $10\;\mathrm{kJ}\;{\mathrm{mol}}^{-1}$. The three AIM derived descriptors (ELF, LIE, LED) yielded very similar results (Figure 7) but most values overestimate the barrier. We conclude that the IBSI is best suited for the characterization of the bond breaking and was therefore used for further studies. In general, it seems that a cutoff of 2% yields excellent agreement with experimental data for the IBSI descriptor.

### 3.4 Benchmark Against Computational Reference Data

**FIGURE 8:**
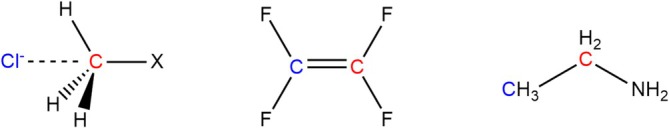
Chemical structure of a difluorocarbene dimer $C_{2}F_{4}$ (left), S_*N*_2‐Encounter complexes $\left\{{\mathrm{Cl}}^{-}{\mathrm{CH}}_{3}X\right\}$ with $X=\left[\mathrm{Cl},\mathrm{Br}\right]$ (middle) and ethylamine ${\mathrm{CH}}_{3}{\mathrm{CH}}_{2}{\mathrm{NH}}_{2}$ (right). The atoms participating in the bond breaking are colored in red and blue.

To assess how well our cost‐effective method performs against computational gas phase reference data, we investigated four structures with differing bond types (see Figure 8).

**FIGURE 9:**
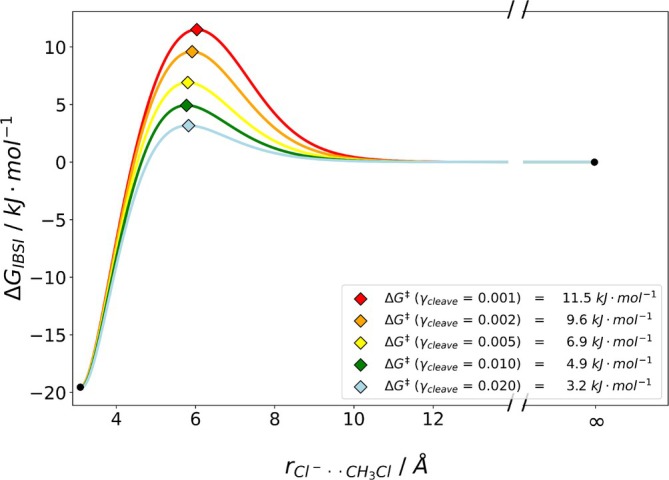
Plot of the estimated dissociation barrier at *T* = 300 K for the ${\mathrm{Cl}}^{-}+{\mathrm{CH}}_{3}\mathrm{Cl}⇌\left\{{\mathrm{Cl}}^{-}\;{\mathrm{CH}}_{3}\mathrm{Cl}\right\}$ encounter complex formation in the gas phase using IBSI as the descriptor. $\gamma_{\text{cleave}}$ value smaller than 0.02 did not result in a barrier. Values were obtained using B3LYP/def2‐TZVP with D4 dispersion corrections at *T* = 300 K.

**FIGURE 10:**
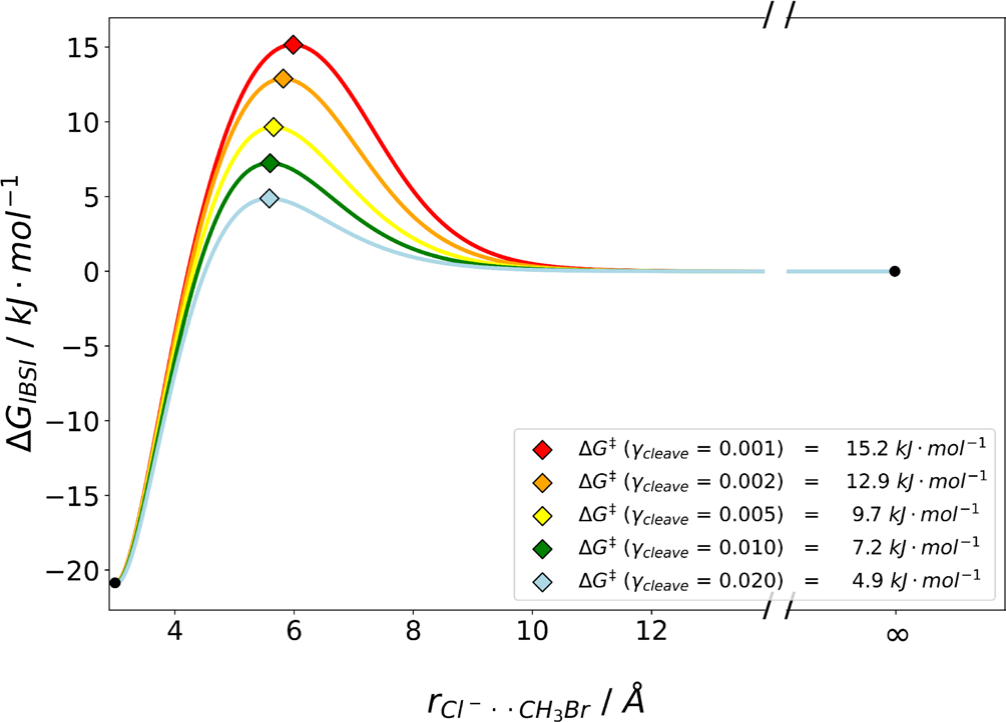
Plot of the estimated dissociation barrier at *T* = 300 K for the ${\mathrm{Cl}}^{-}+{\mathrm{CH}}_{3}\mathrm{Br}⇌\left\{{\mathrm{Cl}}^{-}{\mathrm{CH}}_{3}\mathrm{Br}\right\}$ encounter complex formation in the gas phase using the IBSI descriptor. $\gamma_{\text{cleave}}$ value smaller than 0.02 did not result in a barrier. Values were obtained using B3LYP/def2‐TZVP with D4 dispersion corrections at *T* = 300 K.

First, we investigated the S_*N*_2‐type encounter complex formations for ${\mathrm{Cl}}^{-}+{\mathrm{CH}}_{3}X⇌\left\{{\mathrm{Cl}}^{-}\;{\mathrm{CH}}_{3}X\right\}$ with $X=\left[\mathrm{Cl},\mathrm{Br}\right]$, two systems previously studied by De Sainte Claire et al. [43], who used micro‐canonical variational transition state theory (μCVTST). The results for bond dissociation/association for these weak non‐covalent bonds are depicted in Figure 9 and Figure 10. Values smaller than 0.02 did not results in any reaction barrier. Applying a threshold for $\gamma_{\text{cleave}}$ of 0.02 resulted in barriers of $3.2\;\mathrm{kJ}\;{\mathrm{mol}}^{-1}$ and $4.9\;\mathrm{kJ}\;{\mathrm{mol}}^{-1}$ for the association, in very good agreement with the computational reference values of 3.36 and $3.94\;\mathrm{kJ}\;{\mathrm{mol}}^{-1}$ at 300 K for X = Cl and X = Br, respectively. Notably, our calculated values, while being very small, are within $1\;\mathrm{kJ}\;{\mathrm{mol}}^{-1}$ of the reference value and the range of the accuracy of the applied DFT functional for these systems. Remarkably, our methodology is able to capture the subtle difference in the ${\mathrm{Cl}}^{-}$—C bond in $\left\{{\mathrm{Cl}}^{-}\;{\mathrm{CH}}_{3}\mathrm{Cl}\right\}$ versus $\;\left\{{\mathrm{Cl}}^{-}\;{\mathrm{CH}}_{3}\mathrm{Br}\right\}$.

**FIGURE 11:**
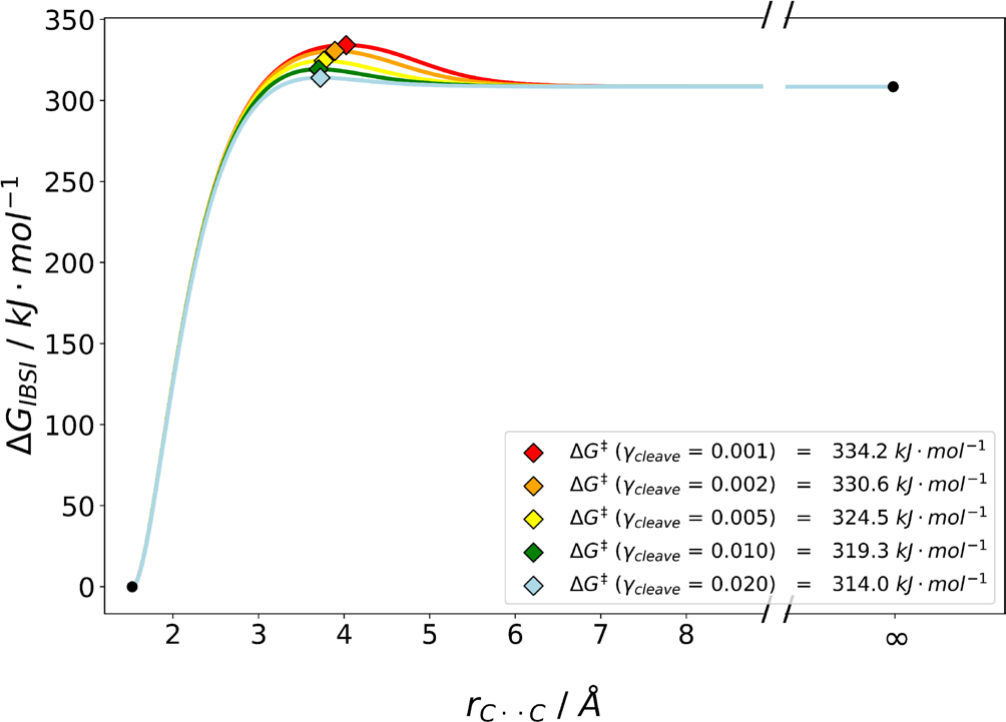
Plot of the estimated dissociation barrier at *T* = 200 K for ${\mathrm{CH}}_{3}{\mathrm{CH}}_{2}{\mathrm{NH}}_{2}⇌{\mathrm{CH}}_{3}+{\mathrm{CH}}_{2}{\mathrm{NH}}_{2}$ in the gas phase the IBSI descriptor. For $\gamma_{\text{cleave}}$ values smaller than 0.02 no barrier was found. Values were obtained using B3LYP/def2‐TZVP with D4 dispersion corrections in a singlet open‐shell (Broken Symmetry) approach at *T* = 200 K.

Next, we were interested in the performance of our algorithm when considering covalent bonds. For the homolytic C—C dissociation in ethylamine, results of our free energy barrier estimation are depicted in Figure 11. Again, $\gamma_{\text{cleave}}$ values smaller than 0.02 did not yield any reaction barrier. For a $\gamma_{\text{cleave}}$ of 0.02, we obtained a barrier of $314.0\;\mathrm{kJ}\;{\mathrm{mol}}^{-1}$ at 200 K, which matches almost perfectly the multifaceted variable reaction coordinate variational transition state theory (VRC‐VTST) with system‐specific quantum Rice–Ramsperger–Kassel theory (SS‐QRRK) computational reference value of $314.2\;\mathrm{kJ}\;{\mathrm{mol}}^{-1}$ at 200 K reported by Zhang et al. [44].

Similarly, we investigated the homolytic cleavage of $C_{2}F_{4}⇌2{\mathrm{CF}}_{2}$, yielding two difluorocarbenes, another dissociation of a C—C covalent bond. In agreement with the computational reference data available, we chose a reaction temperature of 800 K [22].

**FIGURE 12:**
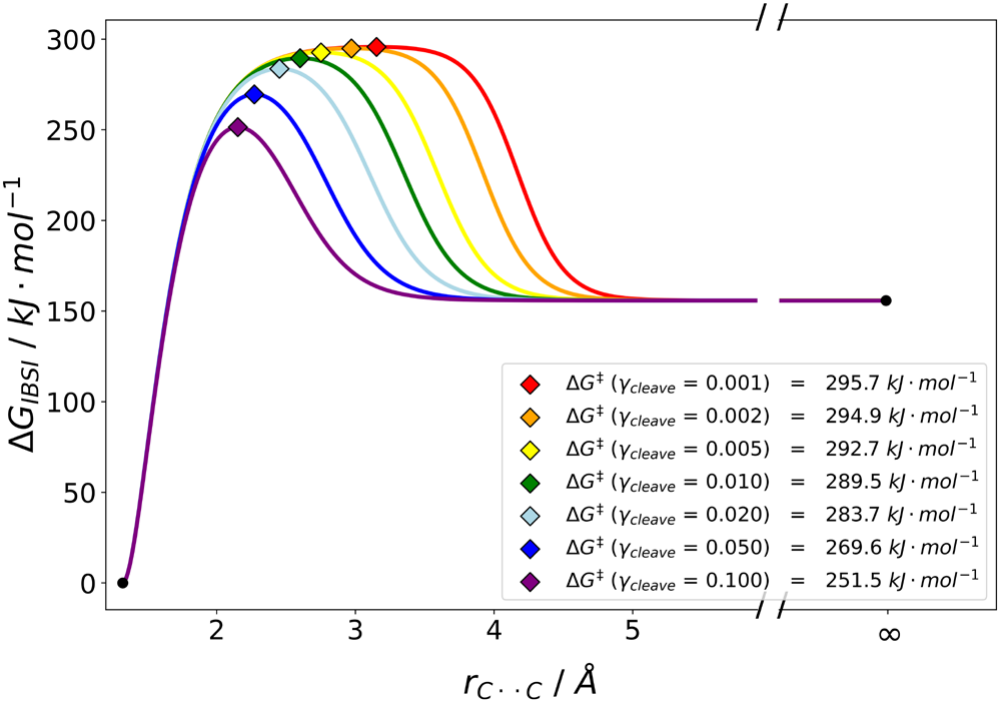
Plot of the estimated dissociation barrier at *T* = 800 K for ${\mathrm{CF}}_{2}$‐dimer using the IBSI descriptor. $\gamma_{\text{cleave}}$ value smaller than 0.02 did not result in a barrier. Values were obtained using B3LYP/def2‐TZVP with D4 dispersion corrections in the gas phase.

The calculated dissociation barrier of $C_{2}F_{4}$ yields values between 251.5 and $295.7\;\mathrm{kJ}\;{\mathrm{mol}}^{-1}$ depending on $\gamma_{\text{cleave}}$ (see Figure 12), while the barrier resulting from VRC‐VTST in combination with SS‐QRRK at this temperature was reported to be at $268.8\;\mathrm{kJ}\;{\mathrm{mol}}^{-1}$ [22]. The best agreement with the reference data is obtained for $\gamma_{\text{cleave}}$ = 0.05 with almost perfect agreement, whereas for our designated cutoff value $\gamma_{\text{cleave}}$ = 0.02, we estimate the barrier to be $283.7\;\mathrm{kJ}\;{\mathrm{mol}}^{-1}$, about $15\;\mathrm{kJ}\;{\mathrm{mol}}^{-1}$ higher than for the reference. Given the large overall barrier, the deviation is small (5%). We also would like to point out here, that the reaction was calculated at high temperature and the accuracy of the RRHO model is uncertain, which could contribute to the difference between the VTST reference data and our methodology. Further inspection of the temperature dependence, revealed that our methodology shows good performance over a large range, but it fails to mimic the increase in barrier height for temperatures above roughly 1200 K (see Figure S8). However, this could potentially be attributed to a breakdown in the RRHO model, where at such high temperatures ro‐vibrational coupling, vibronic excitation, mode coupling and increasing an harmonicity effects play a role. Nevertheless, this example shows that our cost‐efficient methodology compares well with computational benchmarks.

### 3.5 Application to Inorganic Complexes

**FIGURE 13:**
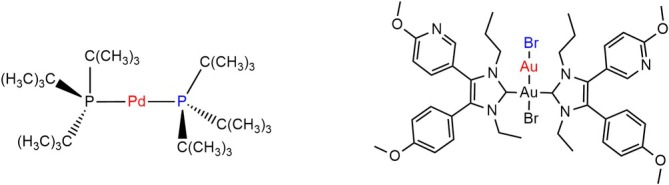
Chemical structure of a ${\mathrm{PdL}}_{2}$ with L = tri‐t‐butylphosphine and $\mathrm{Au}{\left(\mathrm{NHC}\right)}_{2}\text{BrAu}-\mathrm{Br}$ with NHC = diethylbenzimidazol‐2‐ylidene. The atoms participating in the bond breaking are colored in red and blue.

**FIGURE 14:**
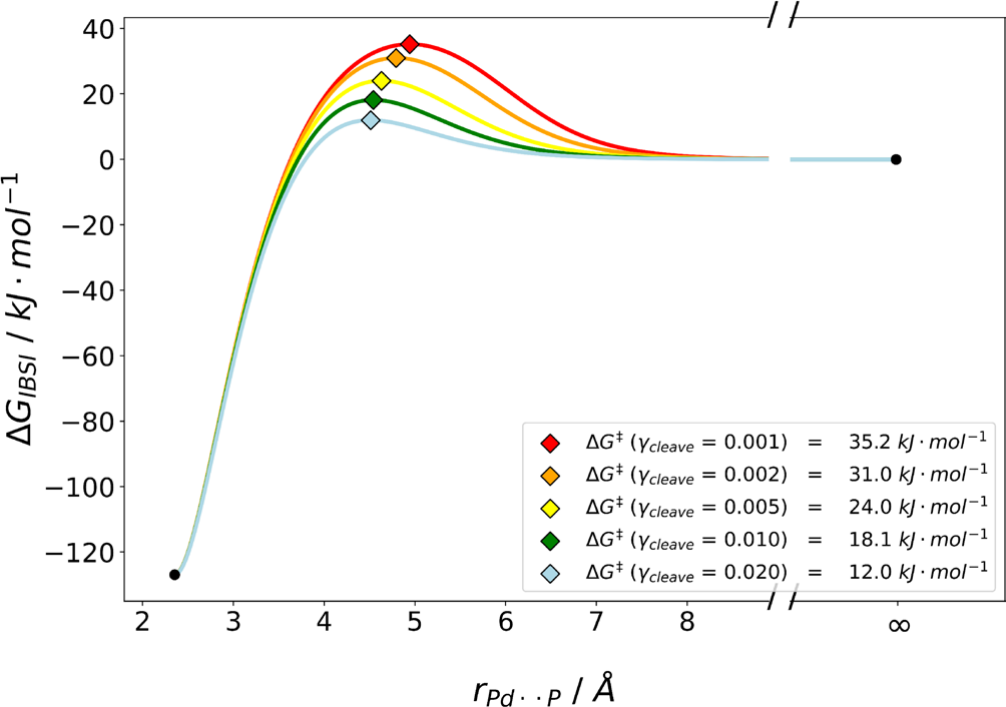
Plot of the estimated dissociation barrier for ${\mathrm{PdL}}_{2}$ complex with the IBSI descriptor. For $\gamma_{\text{cleave}}$‐values below 0.02, no barriers were obtained. Energies were obtained with B3LYP/def2‐TZVP with D4 dispersion corrections and CPCM(toluene).

To further assess the applicability of our methodology, we studied two inorganic complexes: ${\mathrm{PdL}}_{2}$ with L = tri‐*tert*‐butylphosphine and $\mathrm{Au}{\left(\mathrm{NHC}\right)}_{2}\text{BrAu}-\mathrm{Br}$ with NHC = diethylbenzimidazol‐2‐ylidene (see Figure 13). For the first complex, a ligand dissociated from the Pd complex: ${\mathrm{PdL}}_{2}⇌\mathrm{PdL}+L$ [25]. In the second example, a bromide detaches from $\mathrm{Au}{\left(\mathrm{NHC}\right)}_{2}\text{BrAu}-\mathrm{Br}$ [28]. The bond of interest is between the atoms of both complexes, colored in red and blue (see Figure 13). In agreement with experiment [25], these complexes have been investigated in implicit solvent. Again, we computed all $\gamma_{\text{cleave}}$ for both complexes. Note, that for these reactions, the association barrier was studied, which is why the fully dissociated complex plus ligand have been set to $0\;\mathrm{kJ}\;{\mathrm{mol}}^{-1}$. The determined Gibbs free energy association curves and barriers for ${\mathrm{PdL}}_{2}$ are depicted in Figure 14.

**FIGURE 15:**
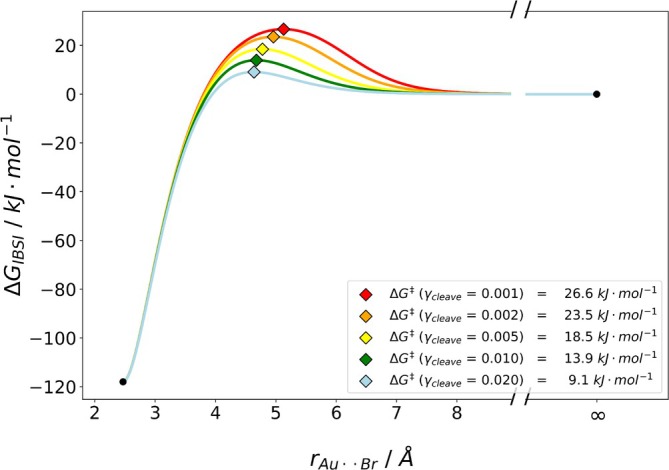
Plot of the estimated dissociation barrier for $\mathrm{Au}{\left(\mathrm{NHC}\right)}_{2}\text{BrAu}-\mathrm{Br}$ with IBSI descriptor. For $\gamma_{\text{cleave}}$‐values smaller than 0.02 no barrier was found. Energies were obtained with B3LYP/def2‐TZVP with D4 dispersion corrections and CPCM(water).

With our methodology, we obtain dissociation barriers of ${\mathrm{PdL}}_{2}$ between 35.2 and $12.0\;\mathrm{kJ}\;{\mathrm{mol}}^{-1}$, while for $\gamma_{\text{cleave}}$ = 0.1, 0.2, and 0.5, no barrier was found. In contrast, the experimentally determined value was ca. $20\;\mathrm{kJ}\;{\mathrm{mol}}^{-1}$, hence, the best agreement was obtained with $\gamma_{\text{cleave}}$ = 0.01. The resulting dissociation surfaces and barriers for $\mathrm{Au}{\left(\mathrm{NHC}\right)}_{2}\text{BrAu}-\mathrm{Br}$ are shown in Figure 15.

The association barrier for the dissociation of ${\mathrm{Br}}^{-}$ was estimated to be between 9.1 and $26.6\;\mathrm{kJ}\;{\mathrm{mol}}^{-1}$, depending on the choice of the $\gamma_{\text{cleave}}$‐value. While $\gamma_{\text{cleave}}$ = 0.1 and 0.05, failed to show a barrier, a $\Delta G^{‡}=13.9\;\mathrm{kJ}\;{\mathrm{mol}}^{-1}$ is determined for $\gamma_{\text{cleave}}$ = 0.01. Consequently, comparing the barriers for both complexes, we obtain $18.5\;\mathrm{kJ}\;{\mathrm{mol}}^{-1}$ versus $13.9\;\mathrm{kJ}\;{\mathrm{mol}}^{-1}$ for the Pd‐ and the Au‐complex, when assuming a $\gamma_{\text{cleave}}$ of 0.01. We see that this methodology captures our chemical intuition, where the bulky phosphine should have a higher barrier than the small ${\mathrm{Br}}^{-}$.

## 4 Discussion

In the scope of this paper, we tested a number of different descriptors including bond order indices and AIM indicators for bond cleavage. While these were shown to be suited for the task at hand, we would like to acknowledge that there are many more quantum chemical descriptors and new ones are continuously being developed. A recent example is the polarizability, recently reported by the Head‐Gordon group [45]. In principle, any quantum chemical descriptor can be used as long as it shows a continuous change in value along the bond dissociation in conjunction with any quantum chemical method. The quality of the estimated entropy curve is highly dependent on the quality of the entropy values for the equilibrium structures. While our methodology is rigorous and straight forward, a remaining challenge is to determine the threshold value $\gamma_{\text{cleave}}$ as it impacts the reaction barrier. Due to the limited dataset, we assume that for gas phase reaction a $\gamma_{\text{cleave}}$ of around 0.02 is adequate at moderate temperatures, while for solution reactions $\gamma_{\text{cleave}}$ = 0.01 is optimal. A deviation for the $C_{2}F_{4}$ can be explained by the higher temperature at which it reacts. The validity of the RRHO‐approximated partition function becomes increasingly questionable at higher temperatures due to effects such as vibrational excitations, mode coupling, and ro‐vibrational interactions. A clear breakdown of the method is observed above 1200 K (Figure S8).

Given that we investigated two distinct sets of molecules, organic molecules in the gas phase vs. transition‐metal complexes in solution, it is reasonable to assume that different $\gamma_{\text{cleave}}$ values emerge. While previous work showed that dynamics of the environment can impact the location of the transition state [46, 47], it is uncertain whether these effects are captured with the polarizable continuum model used in this study. Nevertheless, even a polarizable continuum model affects the electron density distribution of a molecule, which in turn, polarizes the continuum. Hence, its electronic and also geometric structure is altered by the surrounding. Alternatively, the differences in $\gamma_{\text{cleave}}$ values can also arise due to differences in the electronic nature of a transition‐metal–ligand bond. More reference data would be desirable, it is however hard to come by, as diffusion controlled reaction steps are typically not rate determining in multi‐step mechanisms or catalytic cycles. Consequently, there is no straight forward way to measure them and experimental data is very scarce. Similarly, there are very few systems for which computational reference data is reported. However, assessing the values of such diffusion controlled steps becomes increasingly important for microkinetic models. A consistent treatment of diffusion‐controlled steps becomes essential to ensure comparability across similar reactions. In this context, if several barrierless steps appear in a reaction profile, our methodology will capture the differences in the nature of the dissociating ligand. A requirement is that for all diffusion controlled elemental steps the same $\gamma_{\text{cleave}}$ is chosen.

## 5 Conclusion

We have developed a rigorous and cost‐efficient method to estimate the onset of entropy along the reaction coordinate in barrierless reactions. Our methodology is based on standard, static computational chemistry, making use of the rigid rotor harmonic oscillator model to estimate entropic contributions. Assuming that upon bond cleavage, indicated by a quantum chemical descriptor, 50% of the total entropy is obtained, we can unambiguously fit a sigmoid function to model the change in value along the reaction coordinate. Our methodology only requires the expensive calculation of 2^nd^ order derivatives for the complex and the two separated fragments, while the calculation of the quantum chemical descriptor along the reaction coordinate is computationally inexpensive. Comparison with experimental and computational reference data, showed very good agreement and high consistency for $\gamma_{\text{cleave}}$ = 0.02 for gas phase reactions, highlighting the accuracy of our methodology. Investigation of two inorganic complexes showed the applicability to bigger, more complex systems in solution, for which a remarkably similar value of $\gamma_{\text{cleave}}$ = 0.01 was determined. These findings render our methodology a cost‐effective and universally applicable alternative to more intricate methods, such as VTST, for localization of transition states in barrierless reactions.

## Conflicts of Interest

The authors declare no conflicts of interest.

## Supporting information


**Data S1:** jcc70233‐sup‐0001‐Supinfo.pdf.

## Data Availability

The data of the dissociation scans of all structures can be found at the following DOI: https://doi.org/10.5281/zenodo.16782968. The Python3 code in the form of a Jupyter Notebook can be found at the following GitHub repository: https://github.com/PodewitzLab/DORA.git.
