# Supplementary material for: Quantification of Reaction Barriers Under Diffusion Controlled Conditions
Source: J Comput Chem. 2025 Sep 27;46(25):e70233. doi: 10.1002/jcc.70233 (PMC12475963; doi:10.1002/jcc.70233)
Supplement: Supplementary file 1 — Data S1: jcc70233‐sup‐0001‐Supinfo.pdf. [file JCC-46-0-s001.pdf]

Supporting Information

Quantification of Reaction Barriers Under Diffusion Controlled Conditions

Martin M. Maehr,† Radu A. Talmazan,† and Maren Podewitz†,*

E-mail: maren.podewitz@tuwien.ac.at

† Institute of Materials Chemistry, TU Wien, Getreidemarkt 9, 1060 Vienna, AUSTRIA.

Contents

1 Entropic Contributions Using RRHO **2**

2 Additional Descriptors **3**

3 Benchmarking **5**

4 Temperature Dependence of Methodology at the example of C_2F_4 **10**

1 Entropic Contributions Using RRHO

The following figures demonstrate the behaviour of the entropic contributions for the water dimer, when the RRHO is applied to each structure of the dissociation scan,. The total entropic contributions are shown in Figure S1, whereas the vibrational, rotational and translational contributions are depicted in Figure S2.

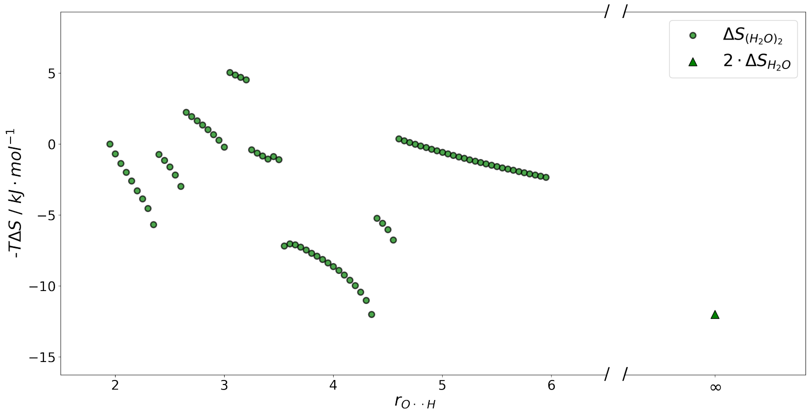

Figure S1: Plot of the nonphysical entropic contributions $-T\Delta S$ during the dissociation reaction when using the Rigid-Rotor Harmonic-Oscillator (RRHO) model for the water dimer. Clearly this approximation is not capable of predicting the entropy for non-equilibrium structures such as a dissociation process. The frequency calculation were obtained using B3LYP [1]/def2-TZVP [2].

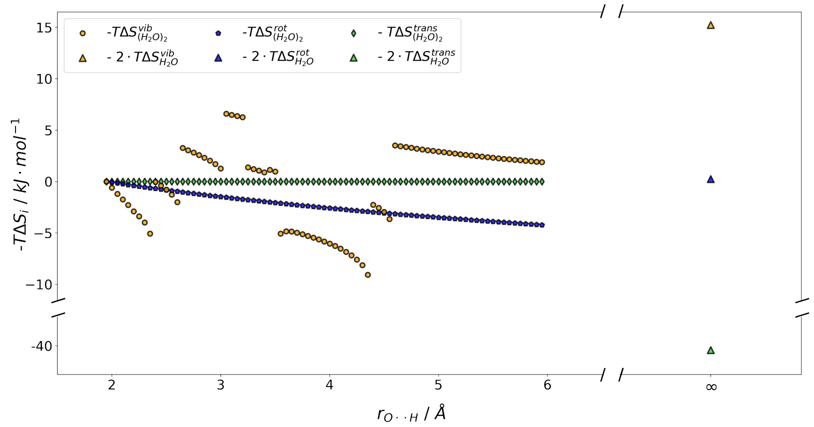

Figure S2: Further investigation of each term of the RRHO contributing to the overall entropy for the water dimer. The orange dots show the vibrational entropy times the temperature, indicating to be the source of the nonphysical bumps in the overall entropy. The frequency calculation were obtained using B3LYP [1]/def2-TZVP [2].

2 Additional Descriptors

Figures S3 and S4 show the results of all additional descriptors that we have investigated in view of their usability for our entropy estimation method at the example of the water dimer.

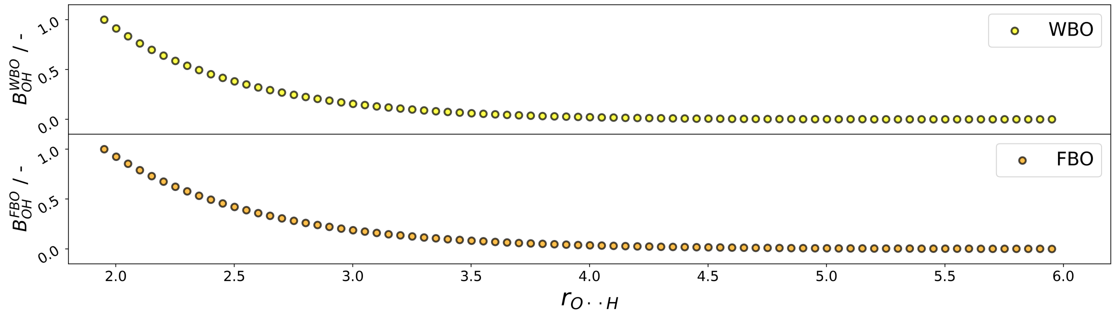

Figure S3: Investigated bond order indices over the intermolecular H-O distance of the water dimer: Wiberg Bond Order [3] (WBO) and Fuzzy Bond Order [4] (FBO). Both bond orders show exponential decrease with varying slopes. All data-point have been normalised.

While we have calculated a number of partial charges (see below), we would like to point out that charges have the conceptual problem that they are reported on two atoms. The same γ_{cleave} may result in two distances and the problem arises as to which one to choose. Hence, they are not well-suited for evaluation. In addition, there are the known difficulties of Mulliken charges: A large dependence on the employed basis set is observed. Large basis sets with more diffuse functions often lead to unphysical results due to the atom-based partitioning scheme. [5] [6]

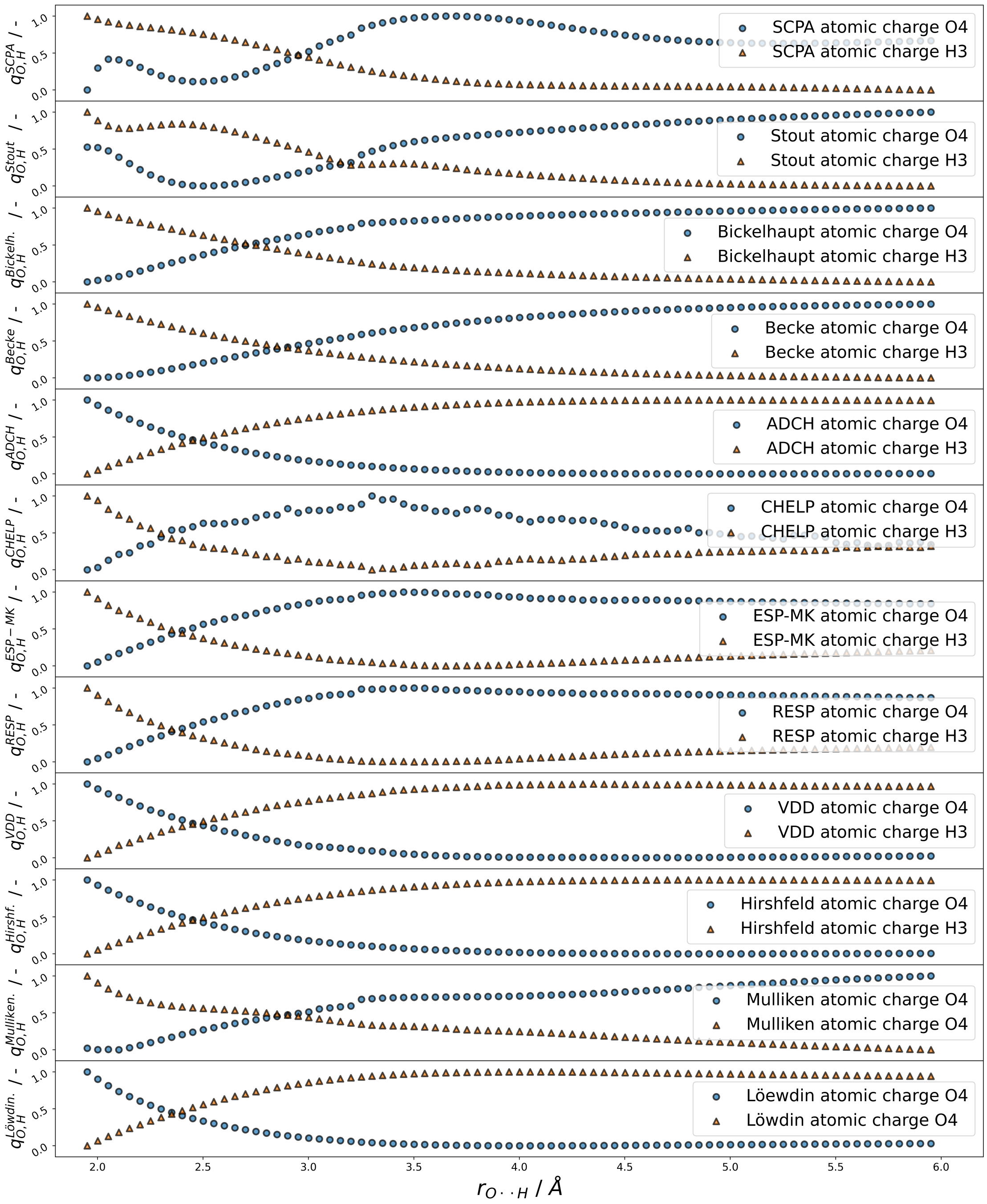

Figure S4: Investigated atomic charges over the intermolecular H-O distance of the water dimer for the oxygen (blue circles) and hydrogen (orange triangles). The investigated atomic charges from top to bottom: Self-Consistent Polarized Continuum Approach [7] (SCPA), Stout–Politzer Charges [8] (STOUT), Bickelhaupt Charges [9] (Bickelhaupt), Becke Population Analysis [10] (Becke), Averaged-Dipole Corrected Hirshfeld Charges [11] (ADCH), CHarges from ELectrostatic Potentials [12] (CHELP) Merz–Kollman Charges [13] (MK), Restrained ElectroStatic Potential Charges [14] (RESP), Voronoi Deformation Density Charges [6] (VDD), Hirshfeld Population Analysis [15] (Hirshfeld) Mulliken Charges [16] (Mulliken) and Löewdin Charges [17] (Löewdin). All data-point have been normalised. The underlying electronic structure has been calculated using B3LYP [1]/def2-TZVP [2].

3 Benchmarking

To study the impact on the dissociation barrier of the water dimer dissociation computed with our methodology, we scompared the two dispersion correction methods, D3 [18] and D4 [19] [20] (see Figure S5). Here, the IBSI descriptor was used. The difference in estimated barrier height values in negligible. Due to the overall better accuracy of D4, we proceeded to calculate the remaining molecules with D4. [20]

Additionally, we investigated the validity of the usage of DFT methods by comparing the electronic energy of the water dimer dissociation energy using B3LYP/def2-TZVP/D3 and CCSD(T) [21]/def2-QZVP. The maximum difference between the two curves is 5 of the order to chemical accuracy (4). Hence, DFT is suited to investigate the systems because more important for us is the difference in barrier (see below).

Using the IBSI to characterize the bond breaking (Figure S6), we obtained the free energy curves. Since it has been shown, that frequencies gained from CCSD(T) calculations perform worse than their cheaper DFT counterparts [22] due to error compensation, we compared the resulting free energy barriers using both the energetics as well as the entropy data gained from CCSD(T)/def2-QZVP, with the barriers that we gain, if we instead use the entropic contributions from DFT methods (B3LYP/def2-TZVP/D3). These results are depicted in Figure S7. For the barriers, which are decisive for our methodology, we observe a difference of roughly 2 (Figure S7 right), a very good agreement.

For extensive benchmarking, computed the dissociation barriers using the functionals BP86 [23], B3LYP [1] [24] and wB97X [25], with the Ahlrichs basis sets [2] def2-SVP and def2-TZVP, with D3 and CPCM [26], in all their permutations using γ_{cleave} sample-set for the following descriptors: IBSI, LBO, FBO, MBO, WBO, ELF, LIE and LED. The resulting barriers are summarized in Table S1 and S2.

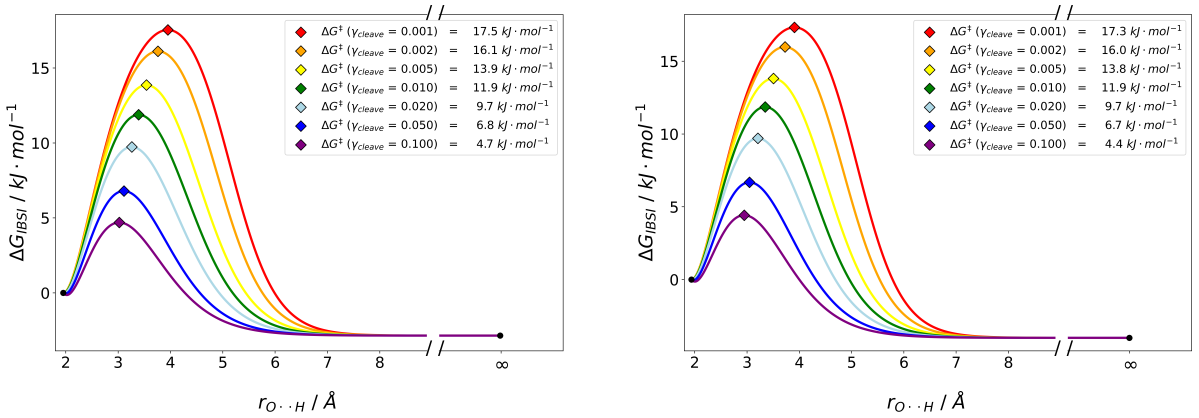

Figure S5: Comparison of ΔG_{fit} and barriers for different different cutoff values γ_{cleave} for the water dimer with the IBSI descriptor using D3 (left) and D4 (right) dispersion corrections. The rainbow coloured curves represent the free energy surfaces, while diamonds indicate estimated barriers. red: $\Delta G(\gamma_{cleave} = 0.001)$; orange: $\Delta G(\gamma_{cleave} = 0.002)$; yellow: $\Delta G(\gamma_{cleave} = 0.005)$; darkgreen: $\Delta G(\gamma_{cleave} = 0.010)$; turquoise: $\Delta G(\gamma_{cleave} = 0.020)$; blue: $\Delta G(\gamma_{cleave} = 0.050)$ violet: $\Delta G(\gamma_{cleave} = 0.100)$. Results were obtained by B3LYP/def2-TZVP with respective dispersion corrections method.

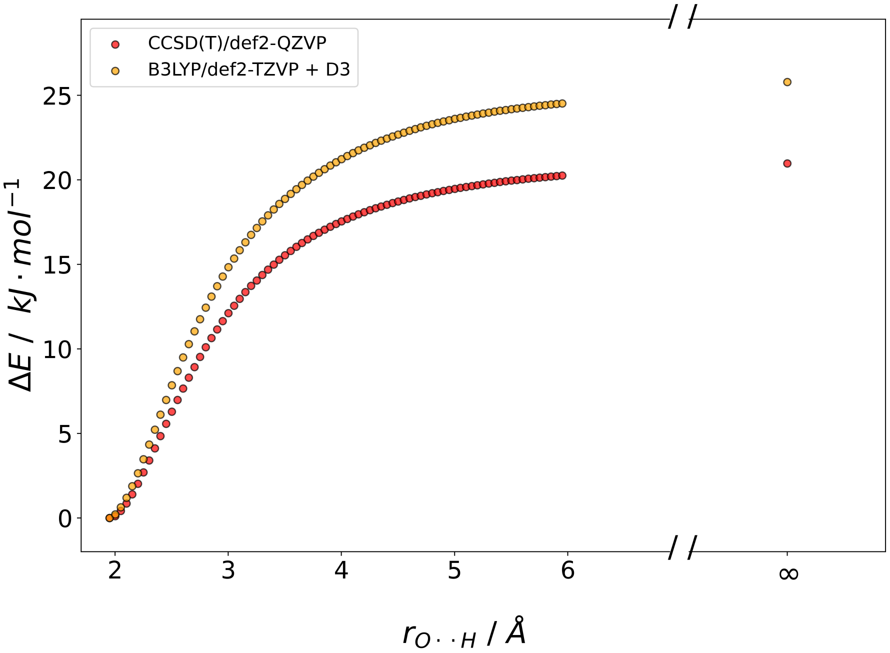

Figure S6: Comparison of the electronic energies for the dissociation reaction of the water dimer using CCSD(T)/def2-QZVP (red dots) and B3LYP/def2-TZVP/D3 dispersion corrections (right). The electronic energy at ∞ correspond to electronic energy of two isolated water molecules.

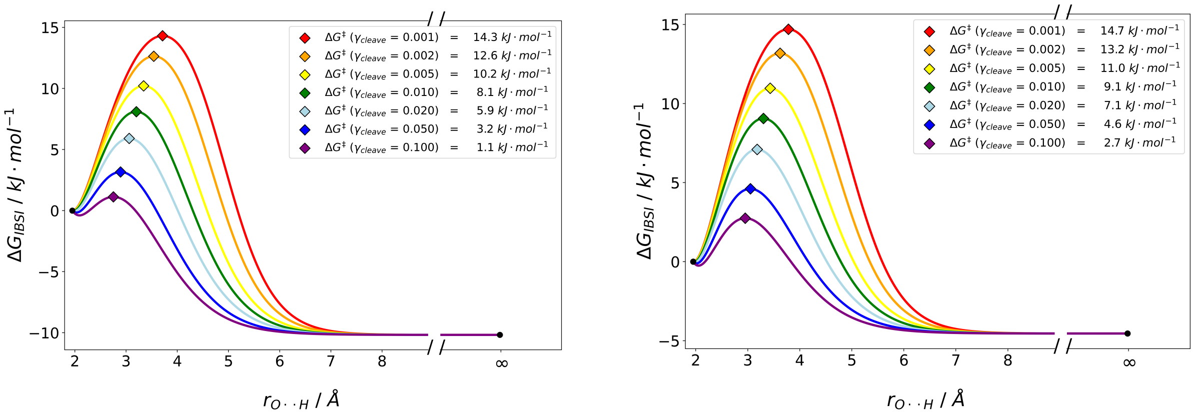

Figure S7: Comparison of ΔG_{fit} and barriers for different different cut-off values γ_{cleave} with the IBSI descriptor for the water dimer using both the electronic energy and the entropic contributions at CCSD(T)/def2-QZVP level of theory (left) and CCSD(T) electronic energies in conjunction with entropy data gained from B3LYP/def2-TZVP/D3 corrections (right) (CCSD(T)/def2-QZVP//B3LYP/def2-TZVP/D3). The estimated transition state are indicated by diamonds. red: $\Delta G(\gamma_{cleave} = 0.001)$; orange: $\Delta G(\gamma_{cleave} = 0.002)$; yellow: $\Delta G(\gamma_{cleave} = 0.005)$; darkgreen: $\Delta G(\gamma_{cleave} = 0.010)$; turquoise: $\Delta G(\gamma_{cleave} = 0.020)$; blue: $\Delta G(\gamma_{cleave} = 0.050)$ violet: $\Delta G(\gamma_{cleave} = 0.100)$.

Table S1: Resulting dissociation barriers for all descriptors using our sample-set for γ_{cleave} for different functionals, basis sets, for gas phase, as well as implicit solvation, dispersion interactions and the combination of solvation and dispersion for the water dimer. All displayed energies are given in kJ mol^{-1}.

Descriptor	γ_{cleave}	B3LYP/def2-SVP				B3LYP/def2-TZVP				BP86/def2-SVP			
		gas	cpcm(h2o)	d3	d3 + cpcm	gas	cpcm(h2o)	d3	d3 + cpcm	gas	cpcm(h2o)	d3	d3 + cpcm
IBSI	0.001	26.4	24.9	29.2	27.3	17.5	14.1	17.5	14.0	25.6	23.9	27.6	26.0
	0.002	24.5	23.9	27.1	26.3	16.1	13.6	16.1	13.6	23.7	23.2	25.6	25.2
	0.005	21.7	22.1	24.1	24.4	13.8	12.6	13.9	12.8	20.7	21.8	22.5	23.5
	0.01	19.5	20.2	21.5	22.5	11.8	11.4	11.9	11.9	18.0	20.2	19.7	21.7
	0.02	17.2	17.9	18.7	20.2	9.6	9.7	9.7	10.5	15.1	18.1	16.6	19.4
	0.05	14.3	14.0	15.0	16.5	6.5	6.3	6.8	8.1	10.9	14.2	12.4	15.2
	0.1	12.5	10.6	12.3	13.4	4.4	3.1	4.7	5.6	7.8	10.7	9.2	11.6
LBO	0.001	18.8	21.2	35.4	23.5	8.3	8.2	8.5	9.4	18.6	20.6	20.2	22.2
	0.002	17.4	19.7	34.6	22.0	7.0	6.7	7.3	8.4	16.6	19.3	18.2	20.7
	0.005	15.5	17.3	32.9	19.7	5.5	4.7	5.8	6.8	13.9	17.1	15.3	18.3
	0.01	14.1	15.2	30.7	17.7	4.4	2.9	4.6	5.5	11.6	15.0	13.0	16.1
	0.02	12.8	12.9	27.8	15.5	3.3	1.3	3.6	4.2	9.4	12.8	10.8	13.7
	0.05	9.3	9.4	22.6	12.3	1.9	0.0	2.3	2.3	6.6	9.2	7.9	10.0
	0.1	9.3	6.8	17.9	10.0	0.9	0.0	1.3	0.9	4.4	6.5	5.8	7.3
FBO	0.001	33.3	26.8	35.4	29.4	21.3	14.8	21.4	14.6	31.0	25.2	33.4	27.6
	0.002	32.4	26.6	34.6	29.2	20.8	14.7	20.8	14.5	30.4	25.1	32.7	27.5
	0.005	30.4	26.2	32.9	28.7	19.4	14.5	19.4	14.4	29.0	24.8	31.2	27.2
	0.01	28.1	25.5	30.7	27.9	17.8	14.2	17.8	14.1	27.1	24.4	29.2	26.6
	0.02	25.2	24.1	27.8	26.5	15.5	13.4	15.6	13.4	24.4	23.4	26.3	25.4
	0.05	20.4	20.9	22.6	23.3	11.5	11.2	11.7	11.7	19.3	20.8	20.9	22.4
	0.1	16.6	17.0	17.9	19.4	8.0	8.0	8.2	9.4	14.3	17.3	15.9	18.5
MBO	0.001	34.4	26.9	36.2	29.5	21.8	14.8	21.8	14.6	31.6	25.2	34.0	27.7
	0.002	34.1	26.8	35.9	29.5	21.7	14.8	21.7	14.6	31.4	25.2	33.8	27.7
	0.005	33.2	26.7	35.2	29.4	21.6	14.8	21.6	14.6	30.9	25.2	33.2	27.6
	0.01	31.8	26.5	34.0	29.1	21.1	14.7	21.1	14.5	29.9	25.0	32.2	27.4
	0.02	29.6	25.9	31.9	28.4	20.1	14.4	20.1	14.3	28.1	24.6	30.2	26.9
	0.05	24.8	23.7	27.1	26.1	17.0	13.3	17.0	13.5	23.7	23.1	25.6	25.0
	0.1	20.1	20.4	21.8	22.7	12.8	10.8	12.9	11.7	18.5	20.4	20.1	21.9
WBO	0.001	33.6	26.8	36.2	29.5	20.8	14.7	20.8	14.5	31.1	25.2	33.5	27.7
	0.002	32.7	26.7	35.9	29.3	20.0	14.7	20.0	14.5	30.6	25.2	33.0	27.6
	0.005	30.9	26.3	35.2	28.9	18.4	14.4	18.4	14.2	29.4	25.0	31.6	27.3
	0.01	28.8	25.7	34.0	28.2	16.5	13.9	16.6	13.8	27.6	24.6	29.7	26.8
	0.02	25.9	24.6	31.9	27.0	14.2	12.9	14.3	13.1	25.0	23.8	27.0	25.8
	0.05	21.1	21.5	27.1	23.9	10.4	10.4	10.5	11.2	19.9	21.5	21.6	23.1
	0.1	17.1	17.6	21.8	20.0	7.1	7.1	7.3	8.7	15.0	18.0	16.5	19.3
ELF	0.001	29.4	25.8	31.6	28.3	20.1	14.1	23.3	16.6	27.7	24.5	29.8	26.8
	0.002	27.9	25.1	29.9	27.6	19.1	14.0	22.1	16.5	26.1	24.1	28.1	26.2
	0.005	25.4	23.7	27.1	26.1	17.2	13.7	19.8	16.1	23.4	23.0	25.2	24.9
	0.01	23.1	22.1	24.4	24.5	15.2	13.3	17.6	15.5	20.8	21.7	22.5	23.4
	0.02	20.4	20.0	21.4	22.4	12.8	12.6	14.9	14.5	17.7	19.8	19.3	21.3
	0.05	16.8	16.2	17.2	18.6	9.1	10.7	11.0	12.0	13.2	16.3	14.7	17.4
	0.1	14.0	12.5	13.9	15.2	6.3	8.4	7.9	9.2	9.7	12.8	11.1	13.7
LIE	0.001	31.9	26.6	34.5	29.2	20.5	14.1	23.3	16.7	30.3	25.1	31.3	27.5
	0.002	30.9	26.3	33.4	28.9	19.6	14.0	22.1	16.6	29.3	24.9	29.9	27.3
	0.005	28.9	25.6	31.1	28.1	17.8	13.9	19.8	16.3	27.4	24.5	27.3	26.7
	0.01	26.8	24.6	28.7	27.0	15.8	13.5	17.6	15.8	25.2	23.7	24.5	25.8
	0.02	24.0	23.0	25.6	25.3	13.5	12.9	14.9	14.9	22.1	22.4	21.2	24.2
	0.05	19.7	19.4	20.5	21.7	9.7	11.3	11.0	12.7	17.0	19.4	16.0	20.8
	0.1	16.1	15.5	16.4	17.9	6.6	9.0	7.9	9.9	12.5	15.7	11.7	16.8
LED	0.001	30.3	26.2	32.6	28.7	18.4	14.0	20.6	16.5	29.1	24.9	31.3	27.3
	0.002	28.8	25.7	31.0	28.2	17.1	13.8	19.0	16.2	27.8	24.6	29.9	26.9
	0.005	26.4	24.5	28.2	26.9	14.9	13.4	16.5	15.6	25.3	23.9	27.3	26.0
	0.01	24.0	23.0	25.4	25.4	12.9	12.8	14.3	14.7	22.7	22.9	24.5	24.8
	0.02	21.2	20.9	22.2	23.3	10.6	11.9	11.9	13.5	19.6	21.2	21.2	22.9
	0.05	17.2	17.0	17.6	19.4	7.4	9.8	8.7	10.8	14.5	17.7	16.0	18.9
	0.1	14.1	13.1	14.0	15.7	4.9	7.3	6.4	8.0	10.3	13.8	11.7	14.8

Table S2: Continuation of the resulting dissociation barriers for all descriptors using our sample-set for γ_{cleave} for different functionals, basis sets, for gas phase, as well as implicit solvation, dispersion interactions and the combination of solvation and dispersion for the water dimer. All displayed energies are given in kJ mol^{-1}.

Descriptor	γ_{cleave}	BP86/def2-TZVP				wB97X/def2-SVP				wB97X/def2-TZVP			
		gas	cpcm(h2o)	d3	d3 + cpcm	gas	cpcm(h2o)	d3	d3 + cpcm	gas	cpcm(h2o)	d3	d3 + cpcm
IBSI	0.001	16.7	13.1	18.9	15.5	29.4	26.5	26.7	24.3	22.0	16.7	19.0	14.3
	0.002	15.4	13.0	17.0	15.3	27.7	25.7	24.9	23.4	20.5	16.5	17.5	14.2
	0.005	13.2	12.7	14.1	14.8	24.8	24.2	21.9	21.8	18.0	16.0	15.0	13.7
	0.01	11.2	12.3	11.6	14.2	22.2	22.5	19.5	20.1	15.8	15.4	12.8	13.2
	0.02	9.0	11.5	8.8	13.2	19.4	20.3	16.7	17.9	13.1	14.4	10.3	12.2
	0.05	6.1	9.7	5.2	10.9	15.4	16.4	13.1	14.0	9.2	12.2	6.6	10.2
	0.1	3.8	7.5	2.6	8.2	12.6	13.0	10.3	10.7	6.1	9.6	3.9	7.8
LBO	0.001	7.5	11.0	7.3	12.3	23.9	23.7	21.3	21.2	11.7	13.9	9.2	11.8
	0.002	6.3	10.3	6.0	11.4	22.0	22.4	19.5	19.9	10.2	12.9	7.7	11.0
	0.005	4.8	9.0	4.0	9.9	19.1	20.1	16.7	17.7	8.1	11.5	5.8	9.5
	0.01	3.7	7.8	2.7	8.4	17.0	18.0	14.7	15.6	6.4	10.1	4.3	8.2
	0.02	2.6	6.3	1.3	6.6	14.8	15.7	12.5	13.2	4.8	8.4	2.8	6.7
	0.05	1.2	4.0	0.0	4.1	11.9	12.0	9.9	9.8	2.8	5.8	1.0	4.4
	0.1	0.3	2.1	0.0	2.2	9.9	9.2	7.9	7.2	1.4	3.7	0.0	2.5
FBO	0.001	20.4	13.2	23.9	15.7	34.4	28.0	32.1	25.9	25.3	16.9	22.7	14.5
	0.002	19.9	13.2	23.1	15.7	33.8	27.9	31.4	25.8	24.8	16.8	22.1	14.5
	0.005	18.6	13.2	21.3	15.6	32.3	27.5	29.6	25.3	23.5	16.8	20.7	14.5
	0.01	17.1	13.1	19.1	15.5	30.4	26.8	27.6	24.6	21.9	16.6	18.9	14.3
	0.02	14.9	12.9	16.2	15.2	27.7	25.7	24.9	23.4	19.5	16.3	16.4	14.0
	0.05	11.0	12.1	11.2	14.0	22.8	22.8	19.9	20.3	15.0	15.1	12.0	12.9
	0.1	7.6	10.6	6.9	12.0	18.2	19.0	15.5	16.6	10.8	13.1	8.1	11.0
MBO	0.001	20.8	13.2	24.9	15.7	35.1	28.1	33.0	26.0	21.8	16.9	23.5	14.5
	0.002	20.7	13.2	24.8	15.7	34.9	28.0	32.7	26.0	21.7	16.9	23.4	14.5
	0.005	20.5	13.2	24.4	15.7	34.3	28.0	32.0	25.9	21.6	16.9	23.1	14.5
	0.01	20.0	13.2	23.6	15.7	33.2	27.7	30.8	25.7	21.1	16.9	22.4	14.5
	0.02	19.0	13.2	21.8	15.6	31.3	27.2	28.8	25.1	20.1	16.8	21.0	14.4
	0.05	15.9	13.0	17.4	15.2	27.0	25.3	24.3	23.0	17.0	16.4	17.4	14.0
	0.1	11.8	12.2	12.0	14.0	22.1	22.3	19.4	19.9	12.8	15.1	12.8	12.8
WBO	0.001	20.0	13.2	23.5	15.7	34.5	28.0	32.3	26.0	25.0	16.9	22.3	14.5
	0.002	19.3	13.2	22.6	15.7	33.9	27.9	31.6	25.9	24.3	16.8	21.5	14.5
	0.005	17.8	13.2	20.6	15.6	32.5	27.6	29.9	25.5	22.9	16.8	19.9	14.4
	0.01	16.0	13.1	18.2	15.4	30.7	27.1	28.0	24.9	21.0	16.6	17.9	14.2
	0.02	13.7	12.9	15.3	15.1	28.0	26.0	25.2	23.7	18.5	16.2	15.4	13.9
	0.05	9.9	12.0	10.4	13.9	23.1	23.3	20.3	20.8	14.0	14.9	11.0	12.6
	0.1	6.7	10.4	6.3	11.8	18.4	19.5	15.8	17.1	9.9	12.8	7.2	10.6
ELF	0.001	19.9	13.2	22.2	15.6	31.5	27.2	28.7	25.0	24.3	16.8	21.3	14.5
	0.002	18.8	13.2	20.8	15.6	30.0	26.7	27.2	24.4	23.3	16.8	20.2	14.4
	0.005	16.8	13.1	18.3	15.4	27.4	25.5	24.5	23.1	21.4	16.6	18.1	14.2
	0.01	14.7	12.9	15.7	15.1	24.9	24.2	21.9	21.7	19.4	16.2	16.0	13.9
	0.02	12.2	12.5	12.8	14.4	22.0	22.2	19.1	19.6	16.7	15.6	13.3	13.2
	0.05	8.3	11.2	8.2	12.7	17.7	18.5	14.9	16.0	12.4	13.9	9.2	11.6
	0.1	5.2	9.3	4.8	10.4	14.2	14.9	11.8	12.4	8.6	11.6	5.9	9.4
LIE	0.001	20.3	13.2	22.8	15.7	33.9	27.9	31.6	25.8	24.7	16.8	22.0	14.5
	0.002	19.4	13.2	21.6	15.6	33.0	27.7	30.5	25.6	23.9	16.8	21.1	14.5
	0.005	17.6	13.1	19.2	15.5	31.1	27.2	28.5	25.0	22.2	16.7	19.3	14.4
	0.01	15.6	12.9	16.8	15.3	29.0	26.3	26.3	24.1	20.3	16.4	17.3	14.1
	0.02	13.1	12.5	13.7	14.7	26.2	25.0	23.3	22.7	17.6	15.9	14.7	13.7
	0.05	9.0	11.2	9.0	13.2	21.2	21.8	18.5	19.4	13.2	14.4	10.3	12.3
	0.1	5.6	9.3	5.3	11.0	17.0	17.9	14.5	15.5	9.3	12.2	6.7	10.2
LED	0.001	18.4	13.2	20.3	15.6	32.4	27.6	30.1	25.6	22.9	16.8	20.3	14.5
	0.002	17.1	13.1	18.6	15.5	31.1	27.2	28.7	25.2	21.6	16.6	18.9	14.4
	0.005	14.8	12.9	15.8	15.2	28.6	26.3	26.2	24.2	19.3	16.3	16.6	14.1
	0.01	12.6	12.6	13.2	14.7	26.2	25.1	23.6	23.0	17.0	15.9	14.4	13.7
	0.02	10.2	12.0	10.4	13.9	23.1	23.3	20.5	21.1	14.3	15.0	11.7	12.9
	0.05	6.7	10.4	6.4	11.9	18.3	19.5	15.9	17.3	10.2	13.0	7.9	11.1
	0.1	4.0	8.4	3.4	9.4	14.6	15.6	12.3	13.5	7.0	10.6	4.9	8.9

4 Temperature Dependence of Methodology at the example of C_2F_4

The Figure S8 compares the resulting barriers of our methodology using different temperatures for our γ_{cleave} sample-set for the difluorocarbene dimer with the resulting barriers using VRC-VTST.[27]

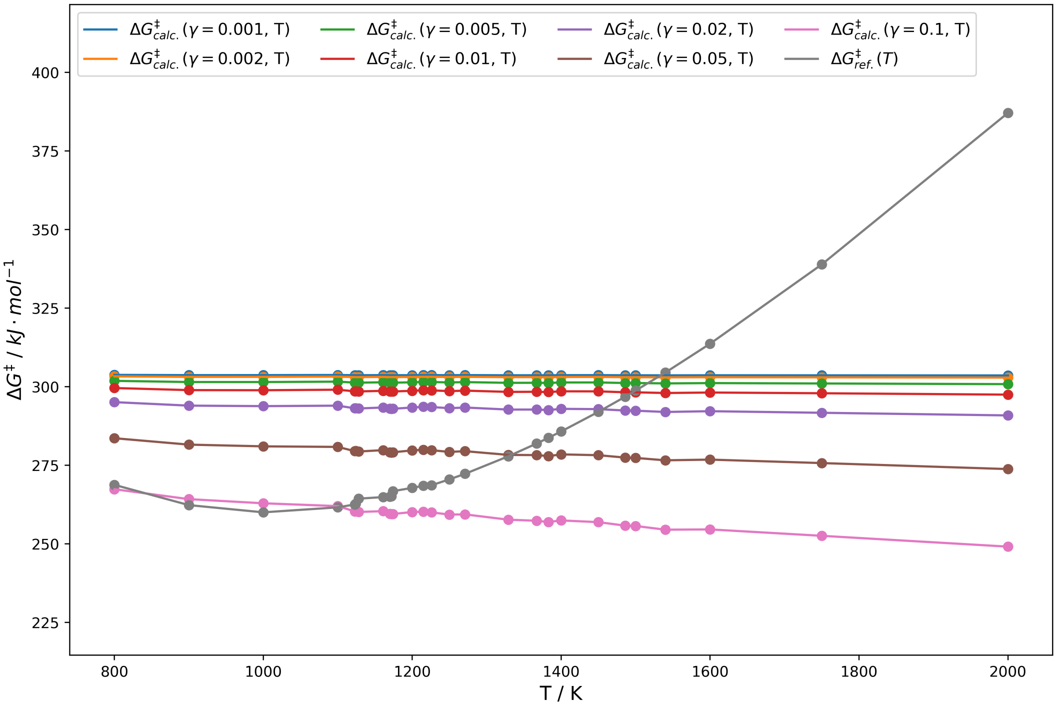

Figure S8: Comparison of the calculated dissociation barriers for C_2F_4 for each cutoff value γ_{cleave} of the sample set for different temperatures using M06L[28]/aug-cc-pVTZ[29] [30] [31]. The grey curve represents the data-computed using VRC-VTST.[27]

References

[1] A. D. Becke, *The Journal of Chemical Physics* **1993**, *98*, 1372–1377.

[2] F. Weigend, R. Ahlrichs, *Physical Chemistry Chemical Physics* **2005**, *7*, 3297–3305.

[3] K. B. Wiberg, *Tetrahedron* **1968**, *24*, 1083–1096.

[4] I. Mayer, P. Salvador, *Chemical Physics Letters* **2004**, *383*, 368–375.

[5] S. C. North, K. R. Jorgensen, J. Pricetolstoy, A. K. Wilson, *Frontiers in Chemistry* **2023**, *11*, 1152500.

[6] C. Fonseca Guerra, J.-W. Handgraaf, E. J. Baerends, F. M. Bickelhaupt, *Journal of Computational Chemistry* **2004**, *25*, 189–210.

[7] P. Ros, G. Schuit, *Theoretica Chimica Acta* **1966**, *4*, 1–12.

[8] E. Stout Jr, P. Politzer, *Theoretica chimica acta* **1968**, *12*, 379–386.

[9] F. M. Bickelhaupt, N. J. van Eikema Hommes, C. Fonseca Guerra, E. J. Baerends, *Organometallics* **1996**, *15*, 2923–2931.

[10] P. Coppens, T. Guru Row, P. Leung, E. Stevens, P. t. Becker, Y. Yang, *Acta Crystallographica Section A: Crystal Physics Diffraction Theoretical and General Crystallography* **1979**, *35*, 63–72.

[11] T. Lu, F. Chen, *Journal of Theoretical and Computational Chemistry* **2012**, *11*, 163–183.

[12] C. M. Breneman, K. B. Wiberg, *Journal of Computational Chemistry* **1990**, *11*, 361–373.

[13] U. C. Singh, P. A. Kollman, *Journal of Computational Chemistry* **1984**, *5*, 129–145.

[14] C. I. Bayly, P. Cieplak, W. Cornell, P. A. Kollman, *The Journal of Physical Chemistry* **1993**, *97*, 10269–10280.

[15] F. L. Hirshfeld, *Theoretica Chimica Acta* **1977**, *44*, 129–138.

[16] R. S. Mulliken, *Journal of the American Chemical Society* **1952**, *74*, 811–824.

[17] P.-O. Löwdin in *Advances in Quantum Chemistry*, *Vol. 2*, Elsevier, **1966**, pp. 213–360.

[18] S. Grimme, J. Antony, S. Ehrlich, H. Krieg, *The Journal of chemical physics* **2010**, *132*, year.

[19] E. Caldeweyher, C. Bannwarth, S. Grimme, *The Journal of chemical physics* **2017**, *147*, year.

[20] E. Caldeweyher, S. Ehlert, A. Hansen, H. Neugebauer, S. Spicher, C. Bannwarth, S. Grimme, *The Journal of chemical physics* **2019**, *150*, year.

[21] J. Noga, R. J. Bartlett, *The Journal of chemical physics* **1987**, *86*, 7041–7050.

[22] D. F. Dinu, M. Podewitz, H. Grothe, K. R. Liedl, T. Loerting, *The Journal of Physical Chemistry A* **2019**, *123*, 8234–8242.

[23] A. D. Becke, *Physical review A* **1988**, *38*, 3098.

[24] J. P. Perdew, *Physical review B* **1986**, *33*, 8822.

[25] J.-D. Chai, M. Head-Gordon, *The Journal of chemical physics* **2008**, *128*, year.

[26] V. Barone, M. Cossi, *The Journal of Physical Chemistry A* **1998**, *102*, 1995–2001.

[27] J. L. Bao, X. Zhang, D. G. Truhlar, *Proceedings of the National Academy of Sciences* **2016**, *113*, 13606–13611.

[28] J. Sun, R. Haunschild, B. Xiao, I. W. Bulik, G. E. Scuseria, J. P. Perdew, *The Journal of chemical physics* **2013**, *138*, year.

[29] T. H. Dunning Jr, *The Journal of chemical physics* **1989**, *90*, 1007–1023.

[30] D. E. Woon, T. H. Dunning Jr, *The Journal of chemical physics* **1993**, *98*, 1358–1371.

[31] K. L. Schuchardt, B. T. Didier, T. Elsethagen, L. Sun, V. Gurumoorthi, J. Chase, J. Li, T. L. Windus, *Journal of chemical information and modeling* **2007**, *47*, 1045–1052.
